# THE REQUIREMENT FOR TRANS AND GENDER DIVERSE YOUTH TO SEEK COURT APPROVAL FOR THE COMMENCEMENT OF HORMONE TREATMENT: A COMPARISON OF AUSTRALIAN JURISPRUDENCE WITH THE ENGLISH DECISION IN *BELL*

**DOI:** 10.1093/medlaw/fwac026

**Published:** 2022-08-02

**Authors:** Malcolm K Smith

**Affiliations:** Griffith Law School, Griffith University, Queensland, Australia

**Keywords:** *Bell*, Children and consent, Family Court of Australia, Gender affirmative hormones, Gender dysphoria, gender diversity, *Gillick* competency

## Abstract

This article outlines the Australian legal position relevant to minors and the commencement of hormone treatment for Gender Dysphoria (GD). It traces the significant Australian legal developments in this field and compares the Australian jurisprudence with recent English caselaw. In *Quincy Bell and Mrs A v The Tavistock and Portman NHS Foundation Trust and Ors*, the English High Court held that minors below 16 years are not likely to have the requisite competency to lawfully consent to the commencement of puberty suppressing drugs. The Court of Appeal subsequently overturned this decision, but there are important aspects of the High Court’s reasoning that warrant further analysis, particularly some of the underlying reasoning about the nature of GD as a condition and its treatment. This article highlights several common themes when comparing the High Court’s reasoning in *Bell* with Australian jurisprudence and highlights how the Australian position has advanced significantly since the first Australian cases in this field were decided. This comparison shows that the Australian perspective is important in demonstrating how judicial views can advance over time alongside a deeper understanding of GD, its treatment, and the broader impact of a requirement to involve the court in such cases. It is concluded that the Australian perspective should be considered in future English cases.

## I. INTRODUCTION

There is a growing body of jurisprudence, discussed throughout this article, that addresses the issue of whether transgender (trans) and gender diverse minors with Gender Dysphoria (GD) are required to seek court approval prior to commencing hormone treatment. These cases have centred on whether court approval is required either as a general safeguard due to concerns about the nature of the treatment, and/or whether the court should confirm the minor’s competency to consent to such treatment. In this article, I outline the Australian case law concerning the requirement for minors to seek court approval for the commencement of hormone treatment for GD and compare it with recent English jurisprudence.

The 5th edition of the Diagnostic and Statistical Manual of Mental Disorders (DSM-5) describes childhood GD as a condition where a child’s subjectively felt identity and gender are not congruent with their biological sex, causing clinically significant distress, or impairment in social functioning or other important areas of functioning.^1^ Treatment for GD in minors can be given in two stages. Stage 1 usually involves the commencement of gonadotropin-releasing hormone agonists referred to as puberty blockers (PBs), which suppress ‘the endogenous oestrogen and testosterone responsible for induction of secondary sexual characteristics’.^2^ In Australia, it is recognised that Stage 1 treatment may be administered to relieve distress ‘for trans adolescents by halting progression of physical changes such as breast growth in trans males and voice deepening in trans females’ and is considered reversible in its effects.^3^

Although other ‘linear growth and weight gain’ continue whilst a minor is on PBs, one key aim is to give them ‘time to develop emotionally and cognitively prior to making decisions on gender affirming hormone use which have some irreversible effects’.^4^ Concerning Stage 2 treatment, the Australian *Standards of Care and Treatment Guidelines for Trans and Gender Diverse Children and Adolescents* explain that:

Gender affirming hormones oestrogen and testosterone are used to either feminise or masculinise a person’s appearance by inducing onset of secondary sexual characteristics of the desired gender. Some of the effects of these medications are irreversible, whilst others have a degree of expected reversibility, that is likely, unlikely or unknown … .^5^

Recent developments in English law relevant to the commencement of PBs in minors stem from the judicial review claim in *Quincy Bell and Mrs A v The Tavistock and Portman NHS Foundation Trust and Ors* (*Bell*).^6^ This was a challenge to the lawfulness of the consent practices adopted by the Gender Identity Development Service (GIDS) at the Tavistock and Portman NHS Trust (Trust) relevant to the commencement of PBs for minors with GD. In December 2020, the High Court concluded that minors below 16 years of age are not likely to have the requisite capacity to lawfully make decisions about the commencement of PBs. This decision was noted to have potentially significant consequences.^7^ In March 2021, *AB v CD & Ors* (*AB v CD*) was decided by the English High Court.^8^ This case focused on whether parents can lawfully consent to the administration of PBs and whether they should be classed as a ‘special’ category of medical treatment necessitating an application to the court in future cases. The High Court held that parents are lawfully able to consent to PBs for the treatment of GD, and that such treatment should not be regarded as ‘special’. Then, in September 2021, the High Court’s decision in *Bell* was overturned by the Court of Appeal.^9^

The aim of this article is to outline the Australian legal position concerning minors and treatment for GD and compare this with the English cases outlined above. Four key themes are identified from the High Court’s judgment in *Bell*. These themes are identified because they share significant overlap with the reasoning expressed in the Australian cases that first addressed the same issue, dating back to 2004. These similarities are explored, together with an overview of how judicial reasoning in Australia has developed since the initial Australian jurisprudence on this topic. The Australian position provides an important point of comparison because, as outlined below, until 2017 there was a general requirement under Australian law to obtain court authorisation for the commencement of Stage 2 treatment,^10^ or for the court to determine the minor’s competency to consent. Furthermore, recent Australian case law suggests an ongoing need for court involvement in some circumstances.^11^ Consequently, there is a significant body of Australian case law that can be compared with the recent English jurisprudence, particularly the reasoning of the High Court in *Bell*, which suggested that court oversight of such decisions was necessary. A central issue in both the Australian and English case law on this topic is the question of whether courts *should* have oversight of decisions about the commencement of hormone treatment for GD. As I discuss, the cases that consider this issue in the respective jurisdictions have analysed the question of whether the court should oversee such decisions using different legal frameworks. In Australia, the law has developed predominantly under the body of law relevant to ‘special medical procedures’, which is focused on parental consent and is outlined in part II. In contrast, the recent English cases have focused mainly on the issue of minors’ competency to make their own medical decisions. Despite the difference in these legal frameworks, the comparison shows many similarities in reasoning between the early Australian case law and the recent English jurisprudence. It also demonstrates that in Australia, there has been a significant shift in judicial thinking on the topic.

In part II, I outline the Australian legal principles relevant to minors and consent to medical treatment, with a particular focus on the law relevant to ‘special medical procedures’. These legal principles are central to the Australian legal framework in this area because they have been relied upon as the basis for the court’s involvement in GD cases. I then provide an overview of the key Australian legal developments specific to minors and hormone treatment for GD. In part III, I outline the recent English decisions on PBs and consent, and summarise the four key themes from these cases which demonstrate a similarity in reasoning with the earlier Australian jurisprudence. The four themes identified from the jurisprudence for this purpose include (i) the view that GD as a condition is unique and that hormone treatment for the condition is unlike other medical treatment, therefore necessitating court oversight; (ii) that minors are not likely to reach a level of competency for the purpose of consenting to Stage 1 and/or Stage 2 treatment; (iii) that decisions about Stage 1 treatment cannot be separated from Stage 2 treatment and the two stages should be considered together; and, (iv) that the seriousness and gravity of the treatment for GD should result in the treatment being regarded as ‘experimental’ or ‘special’. These themes are then used to explore relevant Australian and English jurisprudence, to highlight the similarities, and to show the progression in Australian judicial reasoning. Finally, in part IV, I conclude on these similarities and their significance for future cases, suggesting that the changes in judicial approach in Australia should be considered and adopted in future English decisions, should such cases arise.

## II. THE AUSTRALIAN LEGAL FRAMEWORK

It is well established under Australian law that parents can lawfully consent to medical treatment for their children.^12^ As with the position in England and Wales,^13^ a minor in law is anyone under the age of eighteen.^14^ However, distinct from cases concerning disputes about whether a particular intervention or form of medical treatment is contrary to, or in the best interests of a child, in ‘special medical procedure’ cases the approval of the court is required in *all* cases where the court has previously deemed a specific type of treatment or intervention as ‘special’. Court approval is required in such cases *even if* all parties agree that it is in the best interests of a specific child for the treatment to commence.^15^ The underlying rationale, explained below, is that without court oversight, ‘special’ treatments or interventions carry such a degree of risk about a wrong decision being made by the parents/guardians and potentially treating medical practitioners, that an application to court is necessary to safeguard the best interests of the child.^16^

The landmark Australian High Court decision of *Department of Health and Community Services (NT) v JWB* (*Marion’s Case*)^17^ established the key legal principles relevant to ‘special medical procedures’ in Australia.^18^  *Marion’s Case* is the authoritative decision of the Australian High Court on medical decision-making relevant to children. The case centred on the question of whether Marion’s parents could lawfully consent to her sterilisation. Marion’s parents believed that sterilisation was in the best interests of Marion, who was 14 years of age and profoundly disabled. Sterilisation was proposed as it would help address Marion’s behavioural issues and prevent menstruation and pregnancy. Although the High Court reluctantly drew a distinction between therapeutic and non-therapeutic procedures,^19^ the majority considered that this was necessary in determining whether her parents could lawfully consent to the sterilisation procedure and whether court oversight was required in similar future cases.^20^ The High Court regarded the proposed sterilisation procedure as non-therapeutic because it was for the purpose of preventing menstruation and pregnancy, improving Marion’s hormonal stability, and addressing her behavioural problems, rather than being a ‘by-product of surgery appropriately carried out to treat some malfunction or disease’.^21^ Court oversight was required not only because of the non-therapeutic nature of the procedure, but because this was coupled with a range of further factors. The majority explain:

As a starting point, sterilisation requires invasive, irreversible and major surgery. But so do, for example, an appendectomy and some cosmetic surgery, both of which, in our opinion, come within the scope of a parent to consent to. However, other factors exist which have the combined effect of marking out the decision to authorise sterilisation as a special case. Court authorisation is required, first because of the significant risk of making the wrong decision, either as to a child’s present or future capacity to consent or about what are the best interests of a child who cannot consent, and secondly, because the consequences of a wrong decision are particularly grave.^22^

Although *Marion’s Case* focused on the question of whether non-therapeutic *sterilisation* fell within the parental power to consent, in subsequent cases judges have noted that the categories of special medical procedures are neither closed,^23^ nor limited to sterilisation procedures.^24^ Since *Marion’s Case*, Australian courts have categorised a range of different procedures or interventions as ‘special’, including gender reassignment surgery,^25^ hormone treatment for GD,^26^ surgical intervention on intersex children,^27^ harvesting bone marrow,^28^ and, in Queensland, the termination of pregnancy.^29^ Having said that, not all of these categories are viewed as special medical procedures under current law.^30^

The decision in *Marion’s Case* is also a key authority on the adoption of *Gillick* competency in Australian law.^31^ In *Gillick v West Norfolk and Wisbech Area Health Authority*,^32^ the House of Lords established that a minor can consent to treatment if they are of sufficient maturity and intelligence to understand fully the nature and effect of the decision in question.^33^ As outlined below in part III, the principle of *Gillick* competency is significant in the body of jurisprudence relevant to GD and hormone treatment.

The legal principles set out above have laid the foundations for the legal principles cited in the Australian GD cases. *Marion’s Case* established that a medical intervention is likely to require court approval where it is ‘non-therapeutic’ and where there are further factors, as outlined above, that suggest there is a need to involve the court. This includes where in the absence of court involvement there is a significant risk of making the wrong decision about either the child’s present or future capacity to consent, and about determining the best interests of the child who cannot consent^34^; the procedure in question has particularly grave consequences^35^; the procedure is irreversible and invasive; and there is potential for conflict in terms of the interests of the parties involved; for example, the parents, clinicians, and the patient.^36^

Having set out the foundation, I will now outline the most significant Australian decisions relevant to GD that have considered the need for court oversight, often with reference to the principles from *Marion’s Case*.^37^ The key legal developments summarised here are considered in greater detail in part III, where the decisions in the relevant English cases are explored.

### A. The Starting Point: *Re Alex*

The 2004 first instance decision of the Family Court in *Re Alex: Hormonal Treatment for Gender Identity Dysphoria*^38^ (*Re Alex*), determined that court oversight was required for the commencement of Stage 1 and Stage 2 treatment for gender identity disorder^39^ in minors. As a ward of the state from 10 years of age, Alex was diagnosed with gender identity disorder at thirteen and the Department of Human Services applied to the Family Court for authorisation to proceed with Stage 1 and Stage 2 treatment (authorisation was sought in advance for Stage 2 treatment).^40^ The Family Court was asked to determine, *inter alia*, whether Alex had the capacity to lawfully consent to the treatment and/or whether a parent or guardian could consent to the treatment if Alex lacked the capacity to do so, or whether it was necessary to obtain a court order in such circumstances.^41^

Concerning Alex’s competency, expert evidence before the court outlined that Alex appeared to have a better understanding of the treatment and its associated consequences than that typically expected of a 13-year-old, but the two experts opined that it was not ‘appropriate’ that a person of Alex’s age made the decision to undergo hormone treatment.^42^ Nicholson CJ concluded that as the evidence did not establish that Alex was *Gillick* competent, it was not necessary to reach a conclusion on their capacity. Therefore, the court was required to make a decision based on Alex’s best interests, taking account of their wishes.^43^ Additionally, Nicholson CJ held that Stages 1 and 2 treatment should be considered together as a single treatment package, that it should be classed as a special medical procedure and outside of the parental power to consent. His Honour regarded the treatment as non-therapeutic on the basis that it was for the treatment of a psychiatric disorder rather than treatment of a physical disease or injury.^44^ In addition, Nicholson CJ regarded the treatment as falling within the further factors outlined in *Marion’s Case*: the treatment carried grave, permanent and irreversible consequences (although these factors were noted as applying *only* to Stage 2 treatment),^45^ and there was a risk of making a wrong decision without court oversight.^46^

### B. Post *Re Alex* and the Full Court’s Decision in *Re Jamie*

*Re Alex* was significant because it set out the requirement for court involvement in GD treatment cases.^47^ However, by 2010 the Family Court appeared to question the underlying legal basis for that decision, as well as the view that hormone treatment for GD was non-therapeutic.^48^ Indeed, by 2013, in a number of first instance Family Court decisions it was concluded that Stage 1 treatment should be regarded as therapeutic as the purpose is to treat a psychiatric disorder.^49^ The view that parents could not lawfully consent to Stage 1 treatment had also been questioned on the basis that such treatment was considered reversible and the risk of making a wrong decision by allowing parents to consent was low.^50^ Thus, Murphy J in *Re Sam and Terry* concluded that Stage 1 treatment should be distinguished from Stage 2, as it was not considered grave due to its reversibility and the low risk of error from misdiagnosis.^51^

In 2013, the appeal in *Re Jamie*^52^ was made to the Full Court of the Family Court (Full Court),^53^ focusing on whether continued court oversight was required in respect of both stages of GD treatment. There were three main grounds raised in the appeal, but it was the first ground that was the most significant in shaping the future direction of the law. The first ground was focused on whether treatment for ‘childhood gender identity disorder’ should be regarded as a special medical procedure (and not within the parental power to consent). The Full Court determined that Stage 1 treatment *should* be regarded as therapeutic and, therefore, within the parental power to consent. However, it was held that Stage 2 treatment should require continued court oversight due to its irreversible nature and potentially grave consequences. The Full Court in *Re Jamie* held that minors assessed by treating health professionals as *Gillick* competent *could* make their own decision in respect of Stage 2 treatment, but that an application to court was necessary to confirm the minor’s capacity. If the court confirmed their competency, then the court would have no further involvement.^54^ As discussed below, this resulted in a significant number of applications to the Family Court in circumstances where the primary purpose of the application was to determine the minor’s competency to consent to Stage 2 treatment.

At the time that *Re Jamie* was decided, the Full Court appeared uncomfortable with the prospect of relinquishing oversight of decisions about Stage 2 treatment. Fiona Kelly agrees with this interpretation and has commented that:

one might also speculate that the court, while stating otherwise, continues to believe that treating a transgendered child *is* a special case, warranting judicial oversight. The judgment of Bryant CJ suggests this might be the case when she states that it is “*the nature of the treatment* at stage two [that] requires that the court determine Gillick competence”.^55^

In *Re Jamie*, Bryant CJ concluded that Stage 2 treatment constitutes a special medical procedure because it is irreversible and because of the effects of the treatment.^56^ Referring to the decision of Dessau J at first instance, it was noted that Stage 2 treatment would cause feminisation of the body (including breast growth), stimulate a marked increase in bone mineral density, have effects on growth plates in the long bones which will promote the eventual closure of the growth plates, and would cause Jamie’s height to be about three or four centimetres shorter than if male puberty had been completed.^57^ The Full Court thus decided that Stage 2 treatment *should* continue to require court oversight, including in cases where the minor concerned was determined as being *Gillick* competent.

What is particularly interesting about the outcome in *Re Jamie* is that the legal basis for retaining oversight of Stage 2 treatment was unclear, because the Full Court concluded that Stage 1 treatment for GD is therapeutic and so fell within the parental power to consent, whereas Stage 2 required continued oversight. This raised the question of why the same reasoning about the therapeutic nature of the treatment did not apply to Stage 2 treatment, based on the principles set out in *Marion’s Case*, and whether the legal basis upon which continued court oversight could be justified was flawed.^58^

### C. Decisions after *Re Jamie* and the Full Court’s Decision in *Re Kelvin*

Following the decision in *Re Jamie*, the number of applications to the Family Court increased progressively year-by-year. Between 31 July 2013 (the date that the Full Court handed down its decision in *Re Jamie*) and 16 August 2017 (shortly before the Full Court heard the appeal in *Re Kelvin*),^59^ the Family Court dealt with sixty-three applications either authorising treatment or confirming the minor’s competency to consent to Stage 2 or Stage 3 treatment.^60^ This represents a sharp increase when compared to the small number of applications that came before the Family Court in the 8 years following the decision in *Re Alex*.^61^

The changes adopted by the Full Court in *Re Jamie* were viewed as positive by some commentators,^62^ but as not going far enough by others.^63^ Following the decision in *Re Jamie*, some Family Court judges questioned the need to involve the court in decisions about Stage 2 treatment, and have openly acknowledged in their reasons that the requirement to involve the court was potentially harming rather than protecting children.^64^ For example, in the 2015 decision in *Re Martin*, Bennett J recognised:

the significant emotional toll associated in instituting and participating in any court proceeding not the least part of which is uncertainty around whether there will be a determinative outcome on the day the matter is first listed or if the relief will be granted at all. This is not a burden solely for the parents. It falls to a very great degree on the child and it, doubtless, has been a source of anxiety for him which he could well have done without.^65^

Similarly, Tree J in *Re Lucas*^66^ in 2016, said that there was ‘an urgent need for statutory intervention in order to undo the consequences of *Re Jamie*’, and that the ‘sooner that children such as Lucas and their families do not have to endure the ordeal of litigation in order to get on with their lives, the better’.^67^ At the same time, important legal scholarship was being conducted, which highlighted the possible detrimental impact that the court process was having on young trans and gender diverse persons and their families navigating the court process.^68^ Indeed, the Full Court in *Re Kelvin* acknowledged Professor Fiona Kelly’s work on this point:

In a qualitative study of 12 families … Kelly found that the average delay experienced by those families was eight months from the time that the process was initiated until the adolescent commenced treatment … Fiona Kelly’s 2016 study found the financial costs of the court proceedings varied between those 12 families between $8,000 and $30,000.^69^

In 2017, the Full Court was again asked to clarify the relevant law, this time by way of a case stated.^70^ Following the initial application in *Re Kelvin*, a number of specific questions were referred to the Full Court for determination.^71^ This included the question of whether the Full Court confirmed its earlier decision in *Re Jamie* regarding the requirement that court authorisation should be obtained for Stage 2 treatment and whether it was necessary to continue to make an application to the Family Court to determine a minor’s competency to give consent to such treatment. The joint judgment of Thackray, Strickland, and Murphy JJ (majority judgment) concluded that, generally, Stage 1 and Stage 2 treatment should *not* be subject to court oversight, at least in cases where there is no dispute between the relevant parties concerning the proposed treatment, and that a child’s parents *could* consent to such treatment.^72^ The majority also determined that *Gillick*-competent minors *could* consent to Stage 1 and Stage 2 treatment without the need to have the Family Court confirm the minor’s competency.^73^ The joint judgment of Ainslie-Wallace and Ryan JJ (minority judgment) arrived at the same conclusions, but for different reasons.

The majority in *Re Kelvin* did not go as far as saying that the earlier decision in *Re Jamie* was ‘plainly wrong’.^74^ Instead, the majority identified that there was a factual difference since *Re Jamie* was decided ‘that has relevant legal significance, [such that] there has been a change in the factual understanding on which the earlier decision was based’.^75^ The majority relied on the change in medical understanding and knowledge around GD, including the changes in the DSM-5, as well as the fact that relevant standards had been published in Australia,^76^ and internationally, on childhood GD.^77^ As discussed below, the majority’s reluctance to conclude that the decision in *Re Jamie* was plainly wrong has left in place some aspects of the reasoning from that case.^78^ In contrast, the minority judgment in *Re Kelvin* concluded that the earlier decision in *Re Jamie* was ‘plainly wrong’,^79^ and if this had been the majority opinion that could have changed the course of subsequent judicial developments, not only in relation to GD cases but also to special medical procedures more generally.^80^

### D. Post-*Kelvin* Decisions

Following the Full Court’s decision in *Re Kelvin*, a number of GD cases have been subject to judicial determination in Australia. These cases have involved decisions about Stage 3 treatment, which was not addressed by the Full Court in *Re Kelvin*,^81^ cases of disagreement about whether hormone treatment should commence,^82^ or cases where the consent of both parents for the commencement of treatment could not be obtained.^83^ The most significant of these cases is *Re Imogen (No 6)* (*Re Imogen*), which determined that some aspects of the decision in *Re Jamie* remain binding because the majority of the Full Court in *Re Kelvin* did not conclude that *Re Jamie* was ‘plainly wrong’.

In *Re Imogen*, Watts J determined that applications to the court must occur in circumstances where a parent or medical practitioner disputes: (i) the *Gillick*-competence of the minor; (ii) the diagnosis of GD; and/or (iii) the proposed treatment for GD.^84^ Watts J determined that parental consent is required *in addition* to the consent of the *Gillick* competent minor.^85^ The decision in *Re Imogen* has been noted as a backward step by some commentators, due to the added distress it may cause in circumstances where minors and their families are required to seek the authorisation of the court due to the inability to obtain the consent of both parents, perhaps where one parent is estranged from the family due to a history of family and domestic violence.^86^ Thus, despite the progression in Australian judicial thinking that is highlighted in this article, there are nevertheless problems that remain with the current Australian law on this topic. Interestingly, however, the English first instance decision in *Bell* has also been noted in recent Australian cases, and it has prompted some service providers to review their processes.^87^ Ultimately, however, that decision does not seem to have changed the views of the Australian courts about whether, in principle, a minor may be *Gillick* competent to consent to GD treatment.^88^

## III. RECENT ENGLISH CASE LAW ON GD AND A COMPARISON WITH THE AUSTRALIAN JURISPRUDENCE

The developments outlined above show that the Australian position on GD treatment has changed significantly over time. Thus, the earlier Australian jurisprudence adopted a particularly cautious approach about treatment decisions, not only in relation to the nature of the condition and whether hormone treatment should be regarded as therapeutic, but also about whether a minor could be competent to make decisions about treatment. The English decisions, some of which show a similar approach to the earlier Australian cases, are summarised in this part of the article and the reasoning in these cases is compared with the Australian jurisprudence.

In *Bell*,^89^ two claimants each objected to the consent practices adopted by the GIDS in relation to Stage 1 treatment. They argued that minors are not likely to ever have the capacity to give a legally valid consent to the commencement of PBs. The first claimant was born female and at the age of fifteen started PBs to halt the development of female sexual characteristics. They commenced gender-affirming hormones (testosterone) at the age of seventeen to promote the development of male characteristics, before undergoing surgical intervention as an adult. The claimant regretted this transition and later sought to change their legal sex to reflect their original birth certificate.^90^ The 2nd claimant was concerned that her daughter with autism spectrum disorder, aged fifteen at the time of the claim, might be referred to GIDS and prescribed PBs. The court described the 2nd claimant’s interest in the matter as ‘largely theoretical’.^91^

At first instance, the High Court noted that the GIDS may refer minors to one of two NHS Trusts (the 1st and 2nd interveners in the claim) and that these Trusts may, following referral, provide medical interventions to minors with GD, including PBs. The key legal issue at the heart of the claim focused on whether minors, potentially as young as 10 years of age, might be *Gillick* competent to consent to the commencement of PBs. Under the law of England and Wales, this common law principle applies to minors who are younger than sixteen, as those aged 16 or 17 years are presumed to be competent to consent to their own medical treatment.^92^ The High Court concluded that minors under sixteen were not likely to achieve a level of competency that would allow them to lawfully consent to the commencement of PBs.

As with the position in Australia, under English law, a person who has parental responsibility for a child can ordinarily consent to medical treatment for and on behalf of their non-competent minor in accordance with the best interests of the minor.^93^ However, the issue of whether parental consent *alone* might be sufficient for the purpose of consenting to the administration of PBs in cases where the minor is not *Gillick* competent, did not arise on the facts and was therefore not addressed by the High Court. This was because the practice of the GIDS at the material time was that minors would only be considered eligible to proceed with Stage 1 treatment if they were considered *Gillick* competent. If they were not competent, they were not considered eligible for treatment and so the issue of parental consent did not arise.^94^ This left open the question of whether a minor’s parents could lawfully consent to Stage 1 and/or Stage 2 treatment for GD where the minor is not *Gillick* competent to consent. This is notable because, as I have discussed above, in Australia parents frequently give consent to such treatment in circumstances where the minor is not *Gillick* competent, and the Australian courts have clarified that both Stage 1 and Stage 2 treatment fall within the parental power to consent in such circumstances.^95^

This question of whether a minor’s parents could lawfully consent to Stage 1 and/or Stage 2 treatment for GD where the minor is not *Gillick* competent to consent was the main issue that arose in the subsequent decision of *AB v CD*. In this case, Lieven J concluded that irrespective of whether the minor concerned is *Gillick* competent, parents *can* lawfully consent to Stage 1 treatment.^96^ Lieven J also considered whether PBs should be classed as ‘special’ treatment. It was noted that the reasoning set out by the High Court in *Bell* concerning the nature of the treatment could potentially place it in such a special category, but that ultimately there is no *legal* requirement to involve the court.^97^

The High Court’s conclusions in *Bell* concerning *Gillick* competency were potentially far-reaching. As was subsequently noted by the Court of Appeal:

[the] aim of the litigation was to require … the involvement of the court before anyone under the age of 18 was prescribed [PBs] thus denying the opportunity of consent to such treatment either individually or with the support of their parents or legal guardians.^98^

There are several important aspects of the Court of Appeal’s decision to overturn the High Court’s judgment that merit attention. First, the Court of Appeal noted that the High Court found no illegality in the policy or practice of the defendants,^99^ and, therefore, the declaration that minors are not likely to be *Gillick* competent to consent to PBs extended beyond the immediate legal questions for determination in the judicial review claim. Secondly, the Court of Appeal noted that the issue before the High Court was ‘not a general inquiry into the content of the information and understanding needed to secure the informed consent of a child’, as the legal question was one about the lawfulness of the policy or practice of the defendants.^100^ Furthermore, the Court of Appeal held that many of the High Court’s conclusions were not appropriate in judicial review proceedings and were based on disputed evidence that was not properly tested.^101^ It was also noted that although the High Court’s power to grant declaratory relief or an advisory declaration is broad, the High Court’s approach in *Bell* fell within the dangers outlined by Lord Phillips MR in *R (Burke) v General Medical Council*,^102^ such that the High Court had moved beyond issues relating directly to the parties and, instead, addressed hypothetical questions, laying down propositions that were ‘binding on the world’.^103^

The Court of Appeal thus concluded that:

the effect of the guidance [set out by the Divisional Court] was to require applications to the court in circumstances where the Divisional Court itself had recognised that there was no legal obligation to do so. It placed patients, parents and clinicians in a very difficult position. In practice the guidance would have the effect of denying treatment in many circumstances for want of resources to make such an application coupled with inevitable delay through court involvement.^104^

The outcome of the appeal in *Bell* is welcome, particularly because it establishes that any determination of a minor’s competency falls to treating health professionals with reference to factors established in law.^105^ The Court of Appeal acknowledged that there may be future cases requiring the court to make decisions about the commencement of hormone treatment for GD, due to disagreement between the minor, clinicians, and/or the parents.^106^ Such cases, should they arise, will relate to specific individuals, and the decision of the court will likely focus on the appropriateness of commencing treatment based on whether the minor is competent to make their own decision, and, if not, best interests considerations. Appropriate evidence will need to be provided in such matters to assist the court.

However, as I discuss below, both the Australian case law and the High Court’s judgment in *Bell* demonstrate that judicial views and attitudes towards GD as a condition, its treatment and the minor’s capacity to make decisions, can differ significantly, and these variances can have a profound impact on the outcome in such cases. Although the Court of Appeal makes clear that some of the conclusions reached by the High Court were inappropriate, this conclusion is centred on the scope of the legal issues before the court (in particular, that the matter was a judicial review claim examining the lawfulness of the policy of the GIDS) and that the conclusions were not appropriately based on evidence adduced before the court. It is possible that similar judicial reasoning to that expressed by the High Court may resurface in future cases, supported by appropriate evidence and in circumstances where the scope of the court’s inquiry is not limited in the same way. Whilst the Court of Appeal’s judgment makes clear that such conclusions should be based on evidence, there is, nevertheless, a significant aspect of the judicial reasoning in these cases that is centred on wider issues about the *appropriateness* of such treatment in minors. The Australian jurisprudence provides a particularly important perspective here, as it demonstrates how similarly held and cautious judicial views can change significantly over time, based on an increased understanding of GD, its treatment, and the social context. In fact, the reasoning expressed in the High Court’s judgment shares many similarities with the earlier Australian jurisprudence, which has changed significantly in approach since the initial case of *Re Alex*.^107^ English courts, however, have the benefit of being able to consider how these issues have been determined in other jurisdictions, a luxury that the Australian courts did not have when they first heard this issue.

In reviewing the Australian cases, I consider four key themes from the High Court’s judgment in *Bell*. These themes were central to the High Court’s conclusions in *Bell* and share many similarities to the approach adopted by Australian judges in the earlier case law. I explore the High Court’s reasoning here because the judgment provides an interesting outline of the views and attitudes towards GD as a condition, its treatment, and other wider social and medical issues. While the Court of Appeal’s judgment provides insightful views about the appropriateness of the conclusions based on the evidence, it does not address the underlying views about the condition and its treatment, as expressed in the High Court’s reasons. Nevertheless, as part of the comparison, I also note the impact of the Court of Appeal’s decision in *Bell* concerning each of these themes. The themes from the High Court’s judgment can be summarised as (i) GD as a condition is unique and hormone treatment for the condition is unlike other medical treatment, therefore necessitating court oversight; (ii) minors are not likely to be *Gillick* competent to give consent to Stage 1 and/or Stage 2 treatment; (iii) decisions about Stage 1 treatment cannot be separated from Stage 2 treatment and the two stages should be considered together; (iv) the seriousness and gravity of the treatment for GD should result in the treatment being regarded as ‘experimental’ or ‘special’.

### A. The View that GD and its Treatment Are Unique and Unlike Other Medical Treatment

In *Bell*, the High Court suggested that GD as a condition and together with its treatment, should be regarded as unique.^108^ GD has no ‘direct physical manifestation’ but, and in contrast, treatment for GD has ‘direct physical consequences, as the medication is intended to and does prevent the physical changes that would otherwise occur within the body’.^109^ The High Court questioned whether GD is ‘properly categorised as a psychological condition’, despite it being categorised as such in the DSM-5.^110^ Although the High Court did not further explore whether the categorisation of GD as a condition in the DSM-5 was appropriate,^111^ it was nevertheless concluded that GD *is* different from other conditions:

In other cases, medical treatment is used to remedy, or alleviate the symptoms of, a diagnosed *physical* or *mental* condition, and the effects of that treatment are direct and usually apparent. The position in relation to puberty blockers would not seem to reflect that description.^112^

These views are similar to those outlined in some of the early Australian decisions on GD. In *Re Alex*, for example, Nicholson CJ concluded that treatment for gender identity disorder (as it was then termed under the DSM-IV) is ‘non-therapeutic’, because treatment is not administered to cure a *physical* malfunction or disease of the body but to treat a psychological condition.^113^ Below, I outline three sub-issues that are relevant to the High Court’s views in *Bell* about the nature of the condition and its treatment and highlight how the views in the Australian cases have developed since. This includes reviewing the apparent novelty of the condition and its treatment, the judicial understanding of GD and its treatment, and the role that expert evidence plays in shaping this understanding.

#### 1. The ‘novelty’ of GD and its treatment

Nicholson CJ’s conclusion that gender identity disorder was ‘novel’ and that treatment for the condition should be regarded as non-therapeutic, was central to bringing the treatment within the realms of *Marion’s Case*, and, thereby, classifying it as a special medical procedure necessitating applications to the court in future similar cases.^114^ Hence, Nicholson CJ’s conclusion that treatment is ‘non-therapeutic’ was to engage the precedent established in *Marion’s Case*, thus requiring subsequent decisions about treatment for GD to be referred to the court. The view outlined by the High Court in *Bell* is somewhat more extreme than Nicholson CJ’s approach as the High Court in *Bell* suggests that treatment for GD does *not* constitute treatment for a mental condition.^115^ Thus, this was not a conclusion that was reached for the purpose of bringing treatment decisions concerning GD within an existing legal framework that would require future cases to be referred to the court, although it likely contributed to the view that minors would not be competent to consent to the treatment because of its novelty.

The similarity in reasoning between the first Australian decision and the High Court’s decision in *Bell* might be because of the fact that in both cases the matter had not been previously determined judicially within each respective jurisdiction. Indeed, the High Court in Bell made no reference to other case law that specifically addressed the commencement of treatment in children with GD, particularly the Australian case law, suggesting that the issue was addressed as a truly novel one. Where there is no established authority on a matter, judicial determination of that matter may well result in the conclusion that the issues are novel, and also challenging, as acknowledged by the Court of Appeal in *Bell*.^116^ As was observed by the majority in *Re Kelvin*, decided some 13 years after *Re Alex*:

Nicholson CJ in *Re Alex* found that the application before him ‘would seem a novel one and [he] was not referred to any Australian or overseas authority with similar fact characteristics’. The ‘novel’ application before his Honour and, by inference, the ‘novel’ treatment to which the application referred, shaped Nicholson CJ’s interpretation which, in turn, shaped single-instance judgments thereafter.^117^

In England and Wales, however, the issue in *Bell* was one that had been addressed by a court before. Interestingly, in the 2015 decision of *PD v SD*,^118^ the High Court considered the living arrangements of a 16-year-old who had been placed in foster care and was made a ward of the court. In this case, it was also anticipated that hormone treatment for GD may be provided by the Tavistock Gender Identity Clinic. Although the case was not solely about whether a minor may consent to hormone treatment for GD,^119^ Keehan J held that the minor *was* able to validly consent to medical and surgical treatment in line with section 8 of the Family Law Reform Act 1969. He also upheld the minor’s right to have information kept private about their gender identity and treatment, under article 8 of the European Convention on Human Rights. Notably, Keehan J did not raise any concern about whether the court should be involved in future decisions about hormone treatment. Despite these conclusions, the decision in *PD v SD* was not referred to or relied on by the High Court in *Bell*.

Concerning the apparent ‘novelty’ of the issue, the High Court in *Bell* also did not refer to the significant body of Australian case law on minors and GD. This might be because the strict legal issue before the court in a judicial review claim was different from the types of issue arising in the Australian cases, which concerned applications about treatment decisions concerning specific minors. It might also have been because the parties’ submissions did not refer to the body of Australian case law in this field. However, referring to these cases might have been of use when considering the novelty of the decision and assisted in demonstrating the progression in the judicial understanding of GD as a condition and its treatment. Indeed, unlike Nicholson CJ in *Re Alex*, when *Bell* was heard by the High Court, a significant body of jurisprudence on GD and minors from a common law jurisdiction existed, as discussed in part II above. Indeed, in *AB v CD*, Lieven J noted the Australian position, although this was only in relation to the specific issue of whether PBs should be regarded as a ‘special’ form of treatment and not in relation to the significant progression in the understanding of GD as a condition and its treatment, nor in relation to the other themes identified in this article.^120^

#### 2. Changes in judicial understanding of GD and its treatment

The Australian cases also illustrate how judicial views about GD and its treatment changed after the decision in *Re Alex*. The Full Court in *Re Jamie* heard submissions by the appellants and interveners concerning the unique nature of the condition and its treatment. The public authority made submissions urging the Full Court to distinguish gender identity disorder from other psychiatric disorders, as the pharmaco-therapeutic treatment sought for the condition ‘does not treat the psychological imperative at the heart of the condition, but alters an otherwise healthy body to accommodate to the psychological imperative’.^121^ The public authority also submitted that rather ‘than address a bodily malfunction or disease, the treatment is “inextricably associated with the patient’s self-identity”, in a developmental stage when this is still forming’.^122^ The essence of these submissions is strikingly similar to the views expressed by the High Court in *Bell*. Thus, as outlined above, the High Court in *Bell* views the condition as ‘unique’, particularly because it has no ‘direct physical manifestation’, and observes that treatment for GD has ‘direct physical consequences, as the medication is intended to and does prevent the physical changes that would otherwise occur within the body’.^123^ Such views were rejected by the Full Court in *Re Jamie*, with Bryant CJ commenting that:

The [intervening public] authority submits that the pharmaco-therapeutic treatment sought for childhood gender identity disorder does not treat the psychological imperative at the heart of the condition. However, in my view, that is exactly what it does. If the condition involves self-identity of a different gender from the biological gender with which one is born, then the treatment can be fairly said to address the imbalance of the patient’s self-identity with some, at least, of its bodily representation. In my view, it is not, as the submissions of the public authority propose, the alteration of an otherwise healthy body to accommodate a psychological imperative, but rather it is the alignment of the body with the person’s self-identity.^124^

With reference to this crucial distinction made by Bryant CJ, it should also be highlighted that DSM-5 no longer uses the terminology of gender *identity* disorder, but instead, Gender *Dysphoria*.^125^ As noted by the Full Court in *Re Kelvin*, the change in terminology in the DSM-5 is significant because GD ‘focusses very much on the dysphoria as the disorder to be treated rather than the issue of identity, and identity itself is no longer regarded as any kind of pathology’.^126^ It appears that the High Court in *Bell* exhibited a misunderstanding of the nature of GD. The High Court incorrectly focused on categorising GD as a condition associated with the individual’s gender identity, rather than recognising that the ‘condition’ is, instead, about the dysphoria that results from the incongruence between the individual’s gender and their assigned sex. As Sandra Duffy has noted, the High Court in *Bell* adopted a ‘particularly pathologised’ approach to trans and gender identity.^127^

In addition to the developments in judicial understandings of GD, the Full Court in *Re Kelvin* considered that the state of medical knowledge in 2017 had evolved significantly since the decision in *Re Jamie* in 2013, citing not only the changes in the DSM-5 but also the development of standards of care for the treatment of GD in young persons that existed both in Australia and internationally.^128^ The Full Court in *Re Kelvin* also acknowledged, alongside its observations concerning the advances in the understanding of GD, that one of the main aims of treatment is to alleviate the very significant mental health implications for young persons, including anxiety, depression, self-harm, and attempted suicide.^129^ The Full Court observed that barriers to treatment, such as a requirement to seek court approval, may increase the risk to a minor’s mental health, and that the law might also contribute to minors illegally purchasing hormones to bypass the need for court approval, and recognised that this carries associated risk.^130^ On this basis, it is clear that the Australian jurisprudence examines the broader context relevant to GD and its treatment, and the impact that the legal process can have, and these factors influence the judicial understanding GD and its treatment, and the need to involve the court. This is a point that appears to be missed in some of the English cases. For example, Lieven J’s discussion of ‘special cases’ in *AB v CD* observes that in many of the prior cases concerning other types of medical treatment, where it is concluded that court involvement is *not* justified, the circumstances are such that the minor’s health or condition is potentially fatal or extremely serious.^131^ It is noted in *AB v CD* that the factual, clinical and ethical issues surrounding PBs are different … ‘[i]n particular, the child is not facing a terminal illness, and the treatment has life-changing and life-long consequences, the implications of which are not fully understood’.^132^ However, Lieven J does not acknowledge the very high risk for many young trans and gender diverse persons of self-harm and/or suicide if gender affirming hormone treatment is placed out of reach.

#### 3. Expert evidence and its potential influence on judicial views about GD

The final issue I consider in this section is the role that expert evidence might potentially have in terms of influencing judicial views about the nature of GD as a condition and its treatment. On this, the Australian decision of *Re Imogen*,^133^ decided only months before the High Court handed down its judgment in *Bell*, is interesting as it considered some of the emerging literature that is critical of the gender-affirmative care model for trans and gender-diverse youth. In *Re Imogen*, Watts J considered an application in respect of Imogen (aged sixteen) in circumstances where her parents disagreed about starting Stage 2 treatment. Imogen’s mother fundamentally disagreed with the diagnosis of GD and the conclusions of the treating medical practitioners about Imogen’s capacity to consent to Stage 2 treatment. An expert psychiatrist instructed by Imogen’s mother, referred to as ‘adversarial’ by the Court, disagreed with the idea that GD can be treated with medication under a gender affirming treatment model.^134^ Watts J considered the body of literature adduced in evidence, noting that it:

demonstrates a proliferation of academic and other writings since *Re Kelvin* and the emergence of alternate thinking about treatment and questions arising from the state of knowledge in respect of the long-term implications of current medical treatment for Gender Dysphoria’.^135^

This evidence was analysed in cross-examination and included literature relating to ‘Rapid Onset Gender Dysphoria’.^136^

Watts J outlined that the objections to the gender-affirming treatment model expressed by the mother’s expert psychiatrist were based on a number of concerns. These included: a limited scientific evidence base, underpinned by concerns that it is based on small sample sizes, narrow inclusion criteria, lack of control groups, and lack of longitudinal studies; that there is a lack of research about the physical and psychological long-term effects of hormonal and surgical interventions; and, concerns about regret following medical and surgical transition.^137^ Despite the noted ‘proliferation’ of literature, Watts J rejected many of the expert’s claims in relation to these concerns and refused to accept the expert’s view that Imogen did not have GD.^138^ The Court regarded the position adopted by the Royal Australian College of Physicians (RACP) as important in arriving at this conclusion.^139^ In particular, the Court noted that the RACP supports the position adopted in the Australian guidelines and ‘specifically the emphasis on the multidisciplinary approach to providing person-centred care which prioritises the best interests, preferences and goals of the child or adolescent’.^140^ Similar observations to those raised by the mother and expert in *Re Imogen* were outlined by the claimants in *Bell*. The Court of Appeal expressed concern about the High Court’s reliance on adversarial and untested evidence. The decision in *Re Imogen* may be particularly important for demonstrating that even where such evidence is tested, it may nevertheless be rejected as a basis for regarding the treatment as unique and therefore requiring court oversight. The decision also demonstrates that the court will defer to the relevant healthcare regulatory body for the purpose of determining the efficacy and appropriateness of the treatment in question. A similar approach was noted by the Court of Appeal in *Bell*, when it concluded that disagreements between experts about the nature of GD and its treatment were issues for the NHS to determine as a matter of policy, rather than the courts.^141^

The issues surrounding the nature of the condition and its treatment have been given significant attention in the Australian jurisprudence. Whilst it might appear that a precautionary approach is initially warranted due to the apparent novel nature of the condition and its treatment, Australian judges have retreated significantly from the view that due to the nature of the condition and its treatment, court approval is a necessary safeguard. The Australian courts appear to recognise that the purpose of treatment is to alleviate the significant dysphoria that can arise for young trans and gender diverse persons and that there are potentially very significant health risks that may result should treatment be placed out of reach, including due to the barriers that may result from the need to seek court approval.

### B. The Approach to *Gillick* Competency and the Assumption that a Minor Will Not Be Competent to Consent to Hormone Treatment

In *Bell*, the High Court concluded that minors below 16 years of age are not likely to be *Gillick* competent to consent to PBs. There were several reasons for this conclusion, some of which are discussed in relation to the other themes identified here. One important factor underpinning the Court’s conclusion was the view that the commencement of PBs is a clinical pathway to Stage 2 treatment.^142^ Because of this:

to achieve *Gillick* competence the child or young person would have to understand not simply the implications of taking PBs but those of progressing to cross-sex hormones [CSH]. The relevant information therefore that a child would have to understand, retain and weigh up in order to have the requisite competence in relation to PBs, would be as follows: (i) the immediate consequences of the treatment in physical and psychological terms; (ii) the fact that the vast majority of patients taking PBs go on to CSH and therefore that s/he is on a pathway to much greater medical interventions; (iii) the relationship between taking CSH and subsequent surgery, with the implications of such surgery; (iv) the fact that CSH may well lead to a loss of fertility; (v) the impact of CSH on sexual function; (vi) the impact that taking this step on this treatment pathway may have on future and life-long relationships; (vii) the unknown physical consequences of taking PBs; and (viii) the fact that the evidence base for this treatment is as yet highly uncertain.^143^

The High Court held that a *Gillick* competent minor could consent to PBs *if* they are deemed to be sufficiently mature to understand fully what is involved.^144^ However, the Court concluded that a minor was not likely to reach that threshold with reference to the eight factors outlined above. The Court of Appeal was critical of the High Court’s formulation of the *Gillick* test, noting that both Lord Fraser and Lord Scarman in *Gillick* outlined relevant *guidance* for determining the minor’s competency. For example, Lord Fraser reasoned that:

the doctor will … be justified in proceeding without the parents’ consent or even knowledge provided he is satisfied on the following matters: (1) that the girl (although under 16 years of age) will understand his advice; (2) that he cannot persuade her to inform her parents …; (3) that she is very likely to begin or to continue having sexual intercourse with or without contraceptive treatment; (4) that unless she receives contraceptive advice or treatment her physical or mental health or both are likely to suffer; (5) that her best interests require him to give her contraceptive advice, treatment or both without parental consent.^145^

Lord Scarman added to this, noting that it ‘is not enough that [the minor] should understand the nature of the advice which is being given: [they] must also have a sufficient maturity to understand what is involved’.^146^ After referring to a number of specific factors relevant to the factual circumstances in *Gillick* concerning pregnancy and contraception, Lord Scarman concluded that ‘a doctor will have to satisfy himself that [the minor] is able to appraise these factors before [they] can safely proceed upon the basis that [the minor] has at law capacity to consent to contraceptive treatment.’^147^

The Court of Appeal in *Bell* stated that each of the factors outlined by Lord Scarman ‘was an area for evaluation, rather than a conclusory statement of fact or medical opinion’,^148^ and that some of the eight elements outlined by the High Court did not align with the guidance in *Gillick*.^149^ This included the High Court’s conclusions that the majority of minors who commence PBs proceed to Stage 2 treatment, that there are unknown physical consequences of PBs, and that the evidence base for PBs as a treatment is highly uncertain.^150^

Ultimately, the Court of Appeal held that the guidance outlined by Lords Fraser and Scarman in *Gillick* was not intended to serve as statements of law, as they ‘expressed views about the matters which a clinician would have to explore with a patient, without being prescriptive and recognising that it was for the clinicians to satisfy themselves, in their own way’.^151^ Furthermore, the Court of Appeal held that the High Court ‘was not in a position to generalise about the capability of persons of different ages to understand what is necessary for them to be competent to consent to the administration of puberty blockers’, as it was not deciding a specific case.^152^ The effect of this was to ‘require applications to the court in circumstances where the Divisional Court itself had recognised that there was no legal obligation to do so’.^153^ Consequently, the Court of Appeal held that there is a significant obligation on health professionals to ensure that the minor and their parents ‘appreciate the short and long-term implications of treatment’, and that ‘consent is properly obtained according to the particular individual circumstances … and by reference to developing understanding in this difficult and controversial area.’^154^ Thus, the Court of Appeal suggests that there is a particular need to ensure that the minor (and their parents) understands the implications of the decision to commence treatment for GD, which may require a higher degree of understanding compared to some other treatment decisions due to the consequences of the decision.

Following this, courts in England and Wales may need to address the issue of *Gillick* competency and consent to Stage 1 and/or Stage 2 treatment in future cases should disagreements arise between parents and/or health professionals and young persons. This position is not necessarily different from other decisions about healthcare where there is a dispute between relevant parties such as the clinicians and/or parents. However, what is different is that there is a controversy that remains in relation to treatment decisions for GD, which may therefore impact differently on how the minor’s capacity is assessed. If such matters arise in the future, they will not necessarily be limited to decisions about PBs. Indeed, it is likely that the more significant risks and long-term consequences of Stage 2 treatment will result in a closer analysis of whether a minor has achieved the requisite level of understanding, as has been the situation in Australia, discussed further below. Where disagreements arise, particularly in relation to the minor’s *Gillick* competence, conclusions about their capacity should be made with reference to appropriately tested evidence. However, as the High Court’s judgment in *Bell* demonstrates, there is a risk that some judges may determine that aspects of the treatment are never likely to be fully understood by a minor, due to the nature of the risks and consequences of such treatment. This has also been a concern expressed by some experts and judges in the Australian cases. For example, in *Re Alex*, Nicholson CJ said that:

[i]t is highly questionable whether a 13 year old could ever be regarded as having the capacity [to consent to Stage 1 and Stage 2 treatment], and this situation may well continue until the young person reaches maturity.^155^

This view, in Australia at least, has changed over time and the Australian case law provides some insight into how judges have assessed the *Gillick* competency of minors with GD. In the relevant cases, the courts have relied primarily on medical evidence in order to determine whether the specific minor is, in fact, competent.^156^ Whilst medical evidence may provide the reasons as to why particular minors are considered competent to make decisions about their treatment for GD, the specific factors relevant to the legal determination of the minor’s competency are judicially determined. The specific factors relevant to this determination are not always explained,^157^ but there are several cases where Family Court judges have outlined the factors relevant to assessing the minor’s capacity. In providing an overview of these cases, I start at 2014 because that is the first year after the Full Court’s decision in *Re Jamie*, which determined that the court must confirm the clinical assessment of a minor’s competency. By outlining the applications that have been made to the Family Court for the purpose of seeking approval to commence treatment for GD, together with the outcomes of the relevant decisions, this highlights the change in approach since *Re Alex* in 2004, and demonstrates the willingness of Australian judges to find that minors can reach a level of competency required to make decisions about hormone treatment for GD.

An analysis of the applications made to the Family Court between 2014 and 2017 reveals that out of the seventy-six applications^158^ made during this time that required resolution (noting that some did not require resolution as a result of the decision in *Re Kelvin* in 2017), in sixty-four of these cases the court held that the minors were competent to consent to treatment for GD.^159^ In relation to the remaining twelve applications, the court did not necessarily conclude that the minor lacked *Gillick* competency as, for example, some applications related to minors in foster care and the court was asked instead to consent to Stage 1 treatment.^160^ However, in nine of these seventy-six applications, the court held that the minors lacked the requisite level of understanding to consent to treatment for GD,^161^ and these findings were most frequently based on the views expressed by the clinicians who provided evidence about the minor’s competency. At first glance, the outcomes in these cases demonstrate that Australian judges are willing to find that minors can reach the level of understanding required to make decisions about treatment for GD. As I outline below, some of these cases also demonstrate how the Family Court has approached the issue of *Gillick* competency and the factors that must be assessed to determine the minor’s competency.

In the cases where minors were held to lack the competency to consent to treatment for GD, the court has expressed particular concern about the capacity to fully understand the long-term consequences and unknown risks associated with the treatment. For example, in *Re Karsen* the treating medical practitioner did not believe that Karsen (16 years old) had ‘the capacity to consent to irreversible treatment’ because he had ‘not achieved sufficient understanding of the long term medical consequences to enable him to understand fully what is proposed’.^162^ The expert added that they were ‘not persuaded that most minors would be in the position to fully understand the implications of irreversible hormone treatment over the entire lifespan’,^163^ suggesting that this particular expert may be reluctant to regard minors as *Gillick* competent to consent. The exact same comments were echoed by this expert in a subsequent application later in 2015.^164^ Importantly, despite the expert’s views, the Family Court’s conclusion, in this case, was based on the expert evidence provided, which focused on an assessment of Karsen’s competency to make the decision.^165^ This can be contrasted with the approach of the High Court in *Bell*, which made a global assessment that minors are generally not likely to reach the requisite level of understanding in respect of the long-term issues associated with treatment for GD. Indeed, the Family Court has concluded in other cases that some minors are, in fact, capable of attaining the level of understanding required specifically in relation to the long-term risks and consequences and are consequently *Gillick* competent to make decisions about treatment for GD.^166^

In some of the Australian cases where the minor is deemed *Gillick* competent, the judges helpfully outline their assessment of the minor’s understanding in line with a checklist of factors.^167^ Although, most judges point to medical evidence without explicitly analysing the range of factors relevant to determining the minor’s competency.^168^ This suggests that expert evidence in respect of *Gillick* competency is particularly important and thus, the role that such evidence will play in future cases about treatment for GD should be considered carefully by the court, as outlined above in the previous section.

This ‘checklist’ relevant to assessing *Gillick* competency was first outlined in the decision of *Re Lincoln (No 2)*,^169^ and adopted in *Re Elliott*.^170^ In *Re Elliott*, Tree J observes the utility of the checklist as it ‘usefully emphasises the difference between the child being informed, on the one hand, with being able to process that information into an informed consent, on the other’.^171^ This checklist includes considering the minor’s ability to:

1. Comprehend and retain both existing and new information regarding the proposed treatment;
2. Provide a full explanation, in terms appropriate to the child’s level of maturity and education, of the nature of the treatment;
3. Describe the advantages of the treatment;
4. Describe the disadvantages of the treatment;
5. Weigh the advantages and disadvantages in the balance, and arrive at an informed decision about whether and when to proceed with the treatment;
6. Understand that the decision to proceed with Phase 2 treatment could have consequences that cannot be entirely foreseen at the time of the decision^172^;
7. Understand that the treatment will not necessarily address all or any of the psychological and social difficulties that the person had before the commencement of treatment; and
8. Be free to the greatest extent possible from temporary factors that could impair judgment in providing consent to the treatment.^173^

Although arguably, these factors are not significantly different from those that might ordinarily be considered by a clinician when assessing a minor’s competency to make medical decisions the list has nevertheless been viewed as a useful tool to guide Australian judges in the assessment of *Gillick* competency in such cases, particularly in relation to the long-term risks and consequences.^174^

Importantly, in Australia, the Family Court appears to accept that minors may be capable of understanding the long-term consequences of such treatment. In only a relatively small number of cases, the Family Court has concluded that the minors concerned are not *Gillick* competent, but has nevertheless authorised treatment to commence in line with a best interests assessment in those cases.^175^

What is also evident from the review of the Australian case law is that experts may differ about whether minors may be *Gillick* competent to make decisions about hormone treatment for GD. In this sense, I refer to the global views expressed by some experts, rather than their views about the capacity of specific individuals.^176^ It is this more general question about whether minors can ever be competent to make such decisions that lies at the heart of the High Court’s decision in *Bell*, as appears to be suggested by Lieven J in *AB v CD*.^177^ This issue may well resurface in future cases, and it is one that will require expert evidence for determination. Interestingly, it has recently been suggested that:

there is a clear divergence in practices concerning hormone therapy assessments for trans individuals, with adults having access to the informed consent model of care, while minor youth are still typically required to undergo potentially pathologizing and burdensome mental health evaluations.^178^

As I have outlined in the previous section, the way that some judges approach the nature of GD as condition and its treatment, particularly the view that it is the minor’s gender *identity* that is the ‘condition’ to be treated, is a factor that might contribute to this difference in approach between adults and minors. In their study, Beth Clark and Alice Virani found that ‘youth, parents, and healthcare providers interviewed generally agreed that youth in [the age range of 14 to 18 years of age] can possess the capacity to consent to this care’, and that the determination of the capacity of such persons ‘should be evaluated on an individual basis’.^179^ Although such conclusions are not surprising, they do align with the approach adopted in the Australian cases, and are at odds with the conclusions of the English High Court in *Bell*. Ultimately, in light of the Court of Appeal’s conclusions in *Bell*, English courts will need to make the assessment of *Gillick* competency on an individual basis, with reference to appropriate expert evidence. This requires that it is accepted, at least in principle, that minors may, potentially, be capable of giving consent to Stage 1 and/or Stage 2 treatment for GD.

### C. The View that Stage 1 and Stage 2 Treatment Cannot be Separated and Should be Considered Together as a Single Treatment Decision

In *Bell*, the High Court concluded that it is not possible for a minor to attain a level of competency to consent to Stage 1 treatment without being able to fully understand the impact of all subsequent treatments. For this purpose, a range of factors were examined, such as the reversibility of both Stage 1 and Stage 2 treatment, the treatment pathway (in terms of the longer-term future treatment) once PBs are started, and the long-term impacts and risks of the treatment and interventions that are likely to follow, such as the changes that will result following Stage 2 treatment (beyond the administration of hormone treatment).^180^

The High Court, in effect, viewed the different stages of treatment which are likely to occur many years apart as a single treatment plan that must be understood *prior* to starting the initial stages of treatment. The view that a clear distinction can be made between the stages of treatment was dismissed, because:

evidence shows that the vast majority of children who take PBs move on to take cross-sex hormones, that Stages 1 and 2 are two stages of one clinical pathway and once on that pathway it is extremely rare for a child to get off it.^181^

The Court of Appeal criticised the High Court’s conclusions in this regard. As noted above, the Court of Appeal expressed concern about relying on evidence that was not appropriately tested, and some of this related directly to the High Court’s conclusions about the pathway from Stage 1 to Stage 2 treatment and beyond.^182^ The Court of Appeal noted that in respect of the conclusions made by the High Court about how many minors progressed from Stage 1 to Stage 2 treatment, there was a:

difficulty in drawing conclusions from statistics which are not fully explained or explored in an evidential context where they were peripheral to the legal dispute before the Divisional Court and where any apparent differences were not capable of being tested forensically.^183^

Whilst the Court of Appeal’s conclusion about the nature of the evidence is important, one particular difficulty with the High Court’s approach on this point relates to the underlying assumption that the various stages of treatment should be dealt with together as a single treatment decision. Even if evidence were to establish that a large proportion of minors who commence Stage 1 treatment progress to Stage 2 treatment, this alone is not a basis to determine that at the time the decision to commence Stage 1 treatment is made, this should encompass an understanding of *all* stages of treatment. As discussed below, this is because commencement of Stage 1 treatment is not generally considered to lead automatically to the later commencement of Stage 2 treatment. Thus, the different stages of treatment are typically administered many years apart and are intended to address different stages of the minor’s transition, involving different risks and consequences. For this reason, it is questionable whether a minor must understand the long-term risks and consequences of Stage 2 treatment at the time they commence Stage 1 treatment, as these are distinct treatment decisions.

Initially in Australia, the Family Court grouped Stage 1 and Stage 2 treatment for GD together as a single treatment decision. In *Re Alex*, for example, Alex’s capacity was being assessed in respect of *both* stages of treatment, and this was relevant to Nicholson CJ’s conclusions about this. Nicholson CJ referred to both stages of treatment together as a single ‘treatment package’. Nicholson CJ dealt with both stages of treatment together because it would be ‘destructive and anxiety-provoking’ for Alex if the decision about Stage 2 treatment was left undecided.^184^ However, Australian judges were reluctant to depart from this approach in cases decided after *Re Alex*. Thus, in *Re Brodie*, Carter J said that Stage 1 treatment was the ‘first stage of an overall treatment plan which should be viewed as a single treatment’ package, and agreed with the submission of the Independent Children’s Lawyer in that case, that to view this in any other way would be ‘an artifice’.^185^ There was, however, a change of direction in *Re Jamie*, where the first instance court and the Full Court treated the two stages of hormone treatment separately, and authorised Stage 1 treatment only.^186^ A similar conclusion was reached by the Full Court in 2017 in *Re Kelvin*, and in the minority judgment in particular the significant differences between the two stages of hormone treatment were highlighted.^187^

The view that the two stages of hormone treatment *are* separate can also be found in the relevant clinical standards and guidelines. In the World Professional Association for Transgender Health’s (WPATH) *Standards of Care*,^188^ it is said that the physical interventions for adolescents with GD fall into three stages, that a ‘staged process is recommended to keep options open through the first two stages’, and that ‘[m]oving from one stage to another should not occur until there has been adequate time for adolescents and their parents to assimilate fully the effects of earlier interventions.’^189^ The WPATH *Standards* also state that ‘[p]ubertal suppression *does not* inevitably lead to social transition or to sex reassignment.’^190^ This is significant because the decision about whether Stage 2 treatment is appropriate and whether it should be recommended in a particular case, depends entirely on the individual circumstances of the minor. It necessitates an assessment of how Stage 1 treatment has impacted on the minor’s physical and mental health, whether ongoing treatment is recommended by the treating multi-disciplinary team, and whether that treatment is, in all the circumstances at the material time, in the minor’s best interests.

Even if minors who commence Stage 1 treatment later progress to Stage 2 treatment, this should not create the assumption that starting Stage 1 treatment *always* results in progressing to Stage 2 treatment, and/or that the decision to commence Stage 2 treatment occurs without proper consideration and assessment on the part of the multi-disciplinary team responsible for the minor’s care.^191^ Indeed, the Court of Appeal in *Bell* made it clear that such conclusions should only be made when based on appropriate evidence and that such evidence did not exist to support the High Court’s finding that the vast majority of minors taking PBs go on to Stage 2 treatment and other significant interventions.^192^ Furthermore, the relevant standards and guidelines expect such treatment to be administered under the care of an appropriately qualified multi-disciplinary team.^193^ In Australia, the Full Court in *Re Kelvin* concluded that court oversight of decisions about Stage 2 treatment is not required *only* in circumstances where treatment is provided in accordance with the Australian and international standards/guidelines.^194^ These standards set a clear expectation that at all stages of proposed treatment, the appropriateness of a decision to commence *each stage* of treatment is assessed at the material time in the light of the adolescent’s individual circumstances, as evaluated by the multi-disciplinary team. On this basis, there is an expectation embedded within the relevant professional standards that the process of consent, and therefore, the assessment of the minor’s understanding, will occur at each respective stage of treatment, rather than this occurring for all stages of treatment at the outset, when Stage 1 treatment is commenced.

Reflective of the approach adopted in clinical practice as outlined in the guidelines above, the Australian courts have moved away from the initial view that the stages of treatment for GD should be dealt with as a single treatment decision. Thus, the Australian courts are willing to distinguish between the stages of treatment, meaning that the giving of consent for each stage should be dealt with separately and at the material time that the relevant treatment or intervention is proposed. This may have an important impact on the corresponding capacity that is required in relation to each distinct decision, such that the requisite level of capacity to decide should refer to *each* independent stage of treatment, which can occur many years apart.^195^ This means, for example, that a minor aged eleven may have the capacity to make a decision about Stage 1 treatment at the time such treatment is proposed, but may not have the capacity to consent to Stage 2 at that time. However, by the time a decision about Stage 2 treatment is made, say at 16 years of age (5 years after the initial decision about Stage 1), the minor may have developed a greater level of understanding about what is involved with Stage 2 treatment and its consequences. If, however, the minor was required to understand all aspects of both Stage 1 and Stage 2 treatment at the time the decision about Stage 1 treatment is made, then the minor *may* be less likely to be deemed *Gillick* competent at that earlier point in time. In future cases, the way that the court approaches this issue will potentially impact the assessment of the minor’s capacity.

### D. The View that Treatment Is Experimental and/or ‘Special’

The final aspects of the recent English case law on minors and GD that are significant are the conclusion that Stage 1 treatment should be regarded as experimental, and, in *AB v CD*, the discussion of whether the treatment should be regarded as ‘special’. Although there is a distinction between classifying treatment as ‘experimental’ and categorising it as ‘special’, there is sometimes an important link between these conclusions. Thus, the classification of a form of treatment as experimental *may* provide a justification, in some circumstances, for that treatment to be classed as special, although this is not always the case.^196^ For example, under Australian law, where the intervention in question is not known to have a therapeutic purpose, and the intervention is also considered to carry grave consequences and have irreversible effects, coupled with a risk of making a wrong decision, based on the authority of *Marion’s Case* the intervention may be deemed ‘special’.^197^ In contrast, a procedure or intervention that is experimental but is considered to be ‘therapeutic’, may fall outside of this classification as ‘special’ and not be subject to court oversight.^198^

In *Bell*, the High Court outlined reasons that contributed to the conclusion that PBs should be regarded as experimental treatment:

- there is uncertainty over the short and long-term consequences of the treatment with very limited evidence as to its efficacy, or what it is seeking to achieve^199^;
- there is a lack of clarity over the purpose of the treatment and, in particular, whether it provides a ‘pause to think’ in a ‘hormone neutral’ state or is a treatment to limit the effects of puberty, therefore requiring greater surgical and chemical intervention at a later stage^200^;
- the consequences of the treatment are highly complex and potentially lifelong and are, therefore, life-changing^201^;
- the treatment involves significant long-term and, in part, potentially irreversible long-term physical, and psychological consequences for young persons^202^; and,
- there are currently limited studies/evidence of the efficacy or long-term effects of the treatment.^203^

In particular, the first of these concerns led the High Court to conclude that the treatment should be ‘properly described as experimental treatment’.^204^ This was significant to the conclusion reached because:

the degree to which the treatment is experimental and has, as yet, an unknown impact, does go to the critical issue of whether a young person can have sufficient understanding of the risks and benefits to be able lawfully to consent to that treatment.^205^

The High Court made reference to the *Practice Guidance* relevant to the Court of Protection’s jurisdiction under the Mental Capacity Act 2005.^206^ In particular, paragraph 10 of the *Practice Guidance* makes reference to medical decisions that involve potentially serious interferences with a person’s rights under the European Convention on Human Rights, and paragraphs 11(d) and 11(e) provide specific examples of such interferences, including where treatment is ‘experimental or innovative’ or where the treatment involves ‘a significant ethical question in an untested or controversial area of medicine’, respectively.^207^ Ultimately, the High Court did not rely on this when concluding on the experimental nature of PBs. Nevertheless, when considering whether PBs are a ‘special’ form of treatment in *AB v CD*, although it was determined that they should not be regarded as such,^208^ Lieven J said that:

It is easy to see that arguments might be raised that paragraphs 11(d) and (e) would apply to the administration of PBs for Gender Dysphoria and that therefore the principles applicable to adults lacking capacity should be extended to children.^209^

In relation to the view PBs may be considered ‘experimental’, the Court of Appeal in *Bell* noted that clinical disagreements about issues such as the efficacy of PBs were for the NHS and other relevant bodies to determine as a matter of policy, and not for the courts.

In contrast, Australian judges have not concluded that treatment for GD should be regarded as experimental. However, they have concluded that both stages of treatment, at some time or another, should be classed as special. The reasoning in the Australian cases about why this is the case is similar to the reasoning adopted by the High Court in *Bell*. For example, referring to the reasons of the first instance judge, Bryant CJ in *Re Jamie* noted that Stage 2 treatment for GD has irreversible effects as it causes feminisation of the body (including breast growth), stimulates a marked increase on bone mineral density, and has other significant effects on growth and development, including height.^210^ The Australian courts have acknowledged that Stage 2 treatment is potentially grave and has serious consequences, but have considered these longer-term consequences alongside the therapeutic purpose of the treatment. Thus, the Full Court in *Re Kelvin* said that the gravity of the treatment and its long-term impacts are significant, but that ‘the risks involved and the consequences which arise out of the treatment being at least in some respects irreversible, can no longer be said to outweigh the therapeutic benefits of the treatment’.^211^ Ultimately, the Australian courts have recognised that despite the gravity and uncertainty surrounding Stage 2 treatment, including its long-term effects, these factors no longer provide a justification for classifying the treatment as special or experimental. In *Re Imogen*, Watts J referred to the evidence provided by the RACP in support of this:

the RACP described the treatment for Gender Dysphoria as an emerging area of healthcare where existing evidence on health and wellbeing outcomes of clinical care is limited due to the relatively small number of studies, the small size of study populations, the absence of long-term follow up and the ethical challenges of robust evaluation when control (no treatment) is not acceptable. The College relevantly observes that similar limitations on the existing evidence of healthcare apply to other conditions which affect small segments of the population … .^212^

In Australia, the courts have deferred to the relevant regulatory bodies overseeing the delivery of health care to determine the appropriateness and efficacy of the treatment regime, much like the Court of Appeal’s approach in *Bell*, which held that it is for the NHS to determine matters of policy about whether specific health care interventions should be provided. The Australian cases relevant to treatment for GD have considered the factors that contribute towards the view that hormone treatment should be regarded ‘special’, and some of this reasoning is similar to that adopted by the High Court in *Bell* when concluding that PBs are experimental. Nevertheless, what the Australian cases show is that even in relation to Stage 2 treatment, which is irreversible and carries more serious long-term effects compared to PBs, Australian judges have considered the therapeutic purpose of such treatment and weighed this against the risks and implications of the treatment, concluding that both stages of hormone treatment should no longer be regarded as ‘special’.

## IV. CONCLUSION

In this article, I have explored four key themes that show the similarities in judicial reasoning between the early Australian cases on minors and GD treatment, and the recent English jurisprudence, as well as outlining how Australian judicial reasoning has developed in this area. My detailed examination of the Australian jurisprudence relevant to treatment for GD has demonstrated that although Australian law adopted a different approach to that proposed by the English High Court in *Bell*, the High Court’s reasoning in *Bell* is very similar to that expressed in the early Australian cases. As the Australian position has changed significantly over time, this provides an important point of comparison.

In the recent English cases, the issue of whether the court *should* have oversight of decisions about treatment for GD has been dealt with by way of the law relevant to *Gillick* competence. In contrast, the law in Australia has addressed this question predominantly under the law relevant to ‘special medical procedures’. My analysis of the jurisprudence demonstrates that irrespective of the legal ‘lens’ under which the issues are examined, there are similar concerns and themes that emerge in relation to the nature of GD as a condition, its treatment, and the ability of minors to make decisions about such treatment. The jurisprudence examined in this article demonstrates that the courts have, or at least had, hesitations about allowing minors to commence on a potentially life-altering pathway to affirm their subjectively experienced gender and identity without judicial oversight. The Australian case law not only demonstrates the significant advances in judicial thinking on this topic, but also that Australian courts are now generally accepting of the multi-disciplinary approach to care, supported by relevant regulatory bodies which oversee Australian health professionals. Certainly, the Court of Appeal’s conclusions in *Bell* suggest that questions about the treatment regime for GD are for the NHS to determine, rather than judges. It will be interesting to see how the courts in England and Wales address these issues in the future, should such cases arise, and in particular, whether there is any reference to the Australian jurisprudence in this field.

In the Australian cases, judges have acknowledged that there are significant risks and implications of hormone treatment, but that such risks are outweighed by the therapeutic benefits of allowing young trans and gender diverse persons to access treatment under a multi-disciplinary team without the need to seek court authorisation. Although Australian judges have recognised that GD and the nature of its treatment are different from other types of treatment that the court is asked to make decisions about, this no longer provides a basis to require court oversight in all cases. Moreover, in the Australian decisions, it has been acknowledged that whilst the nature of treatment for GD is serious and there are potential long-term risks and consequences (some of which are uncertain), these are not reasons to regard GD treatment as experimental.

This Australian case law offers an important perspective, and the changes in judicial understanding since the first GD case in 2004 may provide a useful point of comparison for courts in England and Wales when called upon to make decisions of this kind in the future. Although issues remain with the Australian legal position, such as those highlighted following the decision in *Re Imogen*,^213^ it nevertheless provides an example of how judicial understanding and reasoning can develop in a way that is both reflective and responsive to the needs of those that the law is intended to protect.

## Footnotes

1 American Psychiatric Association, *Diagnostic and Statistical Manual of Mental Disorders: DSM-5* (5th edn, American Psychiatric Association Publishing 2013).

2 Michelle Telfer and others, ‘Australian Standards of Care and Treatment Guidelines for Trans and Gender Diverse Children and Adolescents’ (Version 1.3, The Royal Children’s Hospital Melbourne 2020) 15.

3 ibid.

4 ibid.

5 ibid 16.

6 [2020] EWHC 3274 (Admin) (*Bell*).

7 For an overview of the potential impact that this decision would have had see, for example, Kirsty Moreton, ‘A Backwards-Step for *Gillick*: Trans Children’s Inability to Consent to Treatment for Gender Dysphoria—*Quincy Bell & Mrs A v The Tavistock and Portman NHS Foundation Trust and Ors* [2020] EWHC 3274 (Admin)’ (2021) Medical Law Review <https://doi.org/10.1093/medlaw/fwab020> accessed 29 July 2021**;** Peter Dunne, ‘Case and Comment, Childhood in Transition: Can Transgender and Non-Binary Minors Provide Lawful Consent to Puberty Blockers?’ (2021) 80(1) Cambridge Law Journal 15, 16; Abeezar I Sarela, ‘*Bell v Tavistock*: Rethinking Informed Decision-Making as the practical device of consent for medical treatment’ (2021) Clinical Ethics <https://doi.org/10.1177%2F14777509211070499> accessed 30 March 2022; Sinead Helyar and others, ‘Gender Dysphoria in Children and Young People: The Implications for Clinical Staff of the *Bell v Tavistock* Judicial Review and Appeal Ruling’ (2021) Journal of Clinical Nursing <https://doi.org/10.1111/jocn.16164> accessed 30 March 2022; Hannah Hirst, ‘The Legal Rights and Wrongs of Puberty Blocking in England’ (2021) 33(2) Child and Family Law Quarterly 115; Aidan Ricciardo, ‘Minors’ Capacity to Consent to Puberty Supressing Treatment’ (2022) 38(1) Journal of Professional Negligence 38. It has been noted that the decision may also have had an impact beyond decisions about hormone treatment made by trans and gender diverse persons, as it could have applied in the context of non-binary persons who may seek access to PBs for the purpose of halting puberty: Ken C Pang and others, ‘Long-term Puberty Suppression for a Nonbinary Teenager’ (2020) 145(2) Pediatrics e20191606 <https://doi.org/10.1542/peds.2019-1606> accessed 1 August 2021. In this article, I do not analyse the specific issues that may arise in relation to the commencement of PBs by non-binary persons, as the cases to date have not addressed this specific context.

8
*AB v CD & Ors* [2021] EWHC 741 (*AB v CD*).

9
*Quincy Bell and Mrs A v The Tavistock and Portman NHS Foundation Trust and Ors* [2021] EWCA Civ 1363 (*Bell* Court of Appeal).

10 Until 2013, there was also a requirement that Stage 1 treatment was approved by the Family Court of Australia.

11
*Re Imogen (No 6)* [2020] FamCA 761 (*Re Imogen*); for an overview of the circumstances where court approval is required, see Stephanie Jowett and Ben Mathews, ‘Current Legal and Clinical Framework for Treatment of Trans and Gender Diverse Youth in Australia’ (2020) 56(12) Paediatrics and Child Health 1856.

12
*Department of Health and Community Services (NT) v JWB (Marion’s Case)* (1992) 175 CLR 218. The Family Law Act 1975 (Cth) is also relevant, as s 61C provides that each parent of a child under the age of eighteen has parental responsibility for that child (unless altered by an order of a court). s 61B defines parental responsibility to include ‘all the duties, powers, responsibilities and authority which, by law, parents have in relation to children’, which encompasses the authority to make medical decisions on behalf of children. In *Marion’s Case*, the High Court discussed the parental power to consent, but determined that ‘special medical procedures’ do not fall within the powers afforded to parents under the realm of parental responsibility: (Mason CJ, Dawson, Toohey and Gaudron JJ) 263.

13
*Family Law Reform Act 1969* s 1.

14
*Age of Majority Act 1974* (ACT) s 5; *Minors (Property and Contracts) Act 1970* (NSW) s 6; *Age of Majority Act 1974* (NT) s 4; *Law Reform Act 1995* (Qld) s 17; *Age of Majority (Reduction) Act 1971* (SA) s 3; *Age of Majority Act 1973* (Tas) s 3; *Age of Majority Act 1977* (Vic) s 3; *Age of Majority Act 1972* (WA) s 5.

15 Interestingly, this appears to be different to the legal position under English law, where it is acknowledged that the requirement to refer ‘special’ cases to the court exists as a matter of *law*, or whether it exists as a matter of good practice. See, for example, *AB v CD* (n 8) (Lieven J) 71–99.

16 Cameron Stewart, ‘Cracks in the Lintel of Consent’ in Ian Freckelton and Kerry Petersen (eds), *Tensions & Traumas in Health Law* (The Federation Press 2017) 214.

17
*Marion’s Case* (n 12).

18 Cameron Stewart has noted that due to the far-reaching impact of *Marion’s Case*, and the difficulties it has created, particularly in relation to the category of hormone treatment for GD, the decision is ‘a disaster’ as it ‘laid down a test which provided no judicial certainty and it has led to [a situation] where the court’s role is actively causing harm to children’, such that unless there is ‘legislative reform, the High Court urgently needs to rebuild the law of consent for children’: Stewart (n 16) 227.

19 This distinction was also considered, with some reluctance, in the English courts too. See, for example, Lord Hailsham in *Re B (A Minor) (Wardship Sterilisation)* [1987] 2 All ER 206, 213.

20
*Marion’s Case* (n 12) (Mason CJ, Dawson, Toohey and Gaudron JJ) 249–50.

21 ibid. Indeed, the issue of what is regarded as ‘therapeutic’ or ‘non-therapeutic’ is a contentious topic and the reasoning in the High Court decision demonstrates this: see Lesley Naik, ‘When Is the Sterilisation of an Intellectually Disabled Child “Therapeutic”? A Practical Analysis of the Legal Requirement to Seek Court Authorisation’ (2012) 20(2) Journal of Law and Medicine 453.

22
*Marion’s Case* (n 12) 250.

23
*Re Baby D (No 2)* [2011] FamCA 176, [224].

24
*Re Kelvin* [2017] FamCAFC 258, [138].

25
*Re A* (1993) 16 Fam LR 715.

26
*Re Alex: Hormonal Treatment for Gender Identity Dysphoria* [2004] FamCA 297.

27
*Re Lesley (Special Medical Procedure)* [2008] FamCA 1226; *Re Carla* [2016] FamCA 7.

28
*GWW and CMW* (1997) FLC 92-748; *Re Inaya (Special Medical Procedure)* (2007) 38 Fam LR 546.

29
*State of Queensland v B* [2008] QSC 231. Although judicial reasoning about other categories of ‘special’ cases has been criticised, the position in relation to termination of pregnancy has been subject to particular criticism as it is unique in that Queensland is the only State which has required such cases to be referred to the court: see Ben White and Lindy Willmott, ‘Termination of a Minor’s Pregnancy: Critical Issues for Consent and the Criminal Law’ (2009) 17(2) Journal of Law and Medicine 249.

30 For example, the decision in *Re Carla* (n 27) appears to depart from that in *Re Lesley* (n 27), where it was held that surgery on intersex children is a special medical procedure. This decision has been criticised: see, for example, Fiona Kelly and Malcolm K Smith, ‘Should Court Authorisation Be Required for Surgery on Intersex Children? A Critique of the Family Court Decision in *Re Carla*’ (2017) 31(2) Australian Journal of Family Law 118.

31
*Marion’s Case* (n 12) (Mason CJ, Dawson, Toohey and Gaudron JJ) 238–39.

32 [1986] AC 112.

33 In *Marion’s Case* (n 12), the Australian High Court accepted that the principle formed part of Australian law. However, as it was not directly relevant to the decision before the Court, as Marion lacked the capacity in respect of the decision about the sterilisation procedure, the case is *obiter* on that point. Nevertheless, it has been accepted in Australian cases decided by lower courts, including the Full Court of the Family Court of Australia: see, for example, *Re Jamie* [2013] FamCAFC 110; *Re Kelvin* (n 24).

34
*Marion’s Case* (n 12) 250–52.

35 ibid 252.

36 ibid.

37 By significant, I refer here to cases as either decisions of first instance that establish the need for court oversight or decisions at the appellate level, that have determined the need or lack of justification for, the involvement of the courts in such decisions.

38
*Re Alex* (n 26).

39 Note that at that time, the relevant Diagnostic and Statistical Manual of Mental Disorders referred to Gender Identity Disorder, although Nicholson CJ preferred the term Gender Identity ‘Dysphoria’: see American Psychiatric Association, *Diagnostic and Statistical Manual of Mental Disorders* (4th edn, 1990) (‘DSM-IV’). *Re Alex* (n 26) [2].

40 It is interesting to note that this was the first case in Australia that tested whether hormone treatment for GD required the approval of the court as a ‘special medical procedure’, in circumstances where the application arose primarily because of Alex’s status as a ward of the state. The case was not strictly about *parental* decision-making and the application arose because it was the Department of Human Services that was seeking a determination from the Family Court about whether the treatment could be authorised. Later decisions have distinguished the decision for this reason, see *Re Jamie* (n 33) (Bryant CJ) [77]–[78].

41 Another issue for determination in the case was whether the Family Court had jurisdiction to hear the matter: see Fiona Kelly, ‘Australian Children Living with Gender Dysphoria: Does the Family Court Have a Role to Play?’ (2014) 22(1) Journal of Law and Medicine 105, 108.

42
*Re Alex* (n 26) [160] and [164]. This is a concerning position to adopt, given that the key question in the assessment of a person’s decision-making capacity is their ability to understand information relevant to the decision before them, and, in the context of children, the child’s ability to understand fully and to make a decision with maturity. Whether it is *appropriate* for a child to make certain decisions is, arguably, a different value judgement to the question of whether they possess capacity.

43
*Re Alex* (n 26) [169].

44 As noted by Kelly (n 41) 109: ‘[a]fter reviewing the existing case law, none of which dealt with gender dysphoria, Nicholson CJ concluded that it was the intention of the High Court in *Marion’s Case* to limit parental decision-making authority to situations where the child suffers from a disease or malfunctions of *organs*. Therefore, only physical and not psychiatric ailments would meet the test.’

45
*Re Alex* (n 26) [183].

46 ibid [176]–[178].

47 There were a number of applications made to the court in the years following *Re Alex*, including: *Re Brodie* [2008] FamCA 334; *Re Bernadette* [2010] FamCA 94; *Re O* [2010] FamCA 1153; *Re Rosie* [2011] FamCA 63; *Re Jodie* [2013] FamCA 62; *Re Jamie* [2011] FamCA 248; *Re Lucy* (2013) 49 Fam LR 540; *Re Sam and Terry* [2013] FamCA 563.

48 See *Re Bernadette* (n 47), which was appealed to the Full Court in 2011: *Re Bernadette* [2011] FamCAFC 50. The appeal was, however, dismissed because the minor concerned had reached the age of majority and so there was no basis upon which the Family Court could make a decision in respect of an adult.

49
*Re Lucy* (n 47); *Re Sam and Terry* (n 47).

50 ibid. Note that the approach taken in Australian case law surrounding the reversibility of treatment has recently been questioned, due to the nature of the risks and unknown consequences of hormone treatment for GD: Mike O’Connor and Bill Madden, ‘In the Footsteps of Teiresias: Treatment for Gender Dysphoria in Children and the Role of the Courts’ (2019) 27(1) Journal of Law and Medicine 149.

51
*Re Sam and Terry* (n 47) [90].

52 The decision appealed was *Re Jamie* (n 47).

53 The Full Court of the Family Court of Australia hears appeals from decisions made by a single Family Court judge at first instance, or by a Federal Circuit Court judge when the Family Court’s jurisdiction is to be exercised by the Full Court.

54
*Re Jamie* (n 33) (Bryant CJ) [139].

55 Fiona Kelly, ‘Treating the Transgendered Child: The Full Court’s Decision in *Re Jamie*’ (2014) 28 Australian Journal of Family Law 83, 93, emphasis in original, references removed.

56 See *Re Jamie* (n 33).

57 ibid [60], [84].

58 Felicity Bell, ‘Children with Gender Dysphoria and the Jurisdiction of the Family Court’ (2015) 38(2) UNSW Law Journal 426.

59
*Re Kelvin* (n 24).

60 ibid [51]. Stage 3 treatment involves chest reconstructive surgery, also referred to as ‘top surgery’, which ‘may be considered appropriate in the care of trans males during adolescence’: Telfer and others (n 2) 25.

61 The cases determined by the Family Court in the years after *Re Alex* are listed in n 47.

62 Michael Williams and others, ‘Re Jamie (No 2): A Positive Development for Transgender Young People’ (2014) 22(1) Journal of Law and Medicine 90.

63 Kelly (n 55); Bell (n 58).

64 See Stewart (n 16) 226–7.

65 [2015] FamCA 1189 [11].

66 [2016] FamCA 1129.

67 ibid [73].

68 Fiona Kelly, ‘The Court Process Is Slow But Biology is Fast”:* Assessing the Impact of the Family Court Approval Process on Transgender Children and Their Families’ (2016) 30 Australian Journal of Family Law 112.

69
*Re Kelvin* (n 24) [54].

70 The case arose by way of a case stated in the first instance decision of *Re Kelvin* [2017] FamCA 78, referred to the Full Court by Watts J (the judge deciding the original application made by the applicant’s father in respect of a request for authorisation of stage two treatment for GD) made pursuant to s 94A(1) of the *Family Law Act 1975* (Cth).

71 These questions were outlined by Watts J in *Re Kelvin* (n 70).

72
*Re Kelvin* (n 24) [164].

73 ibid [182].

74 ibid [171]–[177].

75 ibid [183].

76 The relevant Australian standards were not yet published but were under development at the time of the hearing in the Full Court decision of *Re Kelvin*. A draft was annexed to the affidavit of Associate Professor Michelle Telfer: *Re Kelvin* (n 24) [9].

77 World Professional Association for Transgender Health, *Standards of Care for the Health of Transsexual Transgender, and Gender Nonconforming People* (version 7, 2012) <https://www.wpath.org/publications/soc> accessed 29 July 2021.

78 See *Re Imogen* (n 11), where it is noted at para 37 that ‘the decision in *Re Kelvin* leaves important parts of what was decided in *Re Jamie* intact.’

79
*Re Kelvin* (n 24) [224].

80 This is noted in *Re Kelvin*, ibid [209].

81
*Re Matthew* [2018] FamCA 161; *Re Ryan* [2019] FamCA 112.

82
*Re Imogen* (n 11).

83
*Re a Declaration Regarding Medical Treatment for “A”* [2020] QSC 389.

84
*Re Imogen* (n 11) [35].

85 ibid.

86 Stephanie Jowett and Fiona Kelly ‘*Re Imogen*: A Step in the Wrong Direction’ (2021) 34(1) Australian Journal of Family Law 31; Fiona Kelly and others, ‘Parental Consent and the Treatment of Transgender Youth: The Impact of *Re Imogen*’ (2022) 216(5) Medical Journal of Australia 219.

87
*Re G2* [2021] FCWA 98 [26]; *Re G3* [2021] FCWA 99 [25]; *Re G4* [2021] FCWA 102 [35].

88 ibid.

89
*Bell* (n 6).

90 ibid [83].

91 ibid [89]. The claimant’s daughter had not ‘been referred to GIDS and having regard to the defendant’s current practice, would not meet the criteria for [puberty blockers] because her parents would not support that treatment’. ibid [89].

92 Family Law Reform Act 1969, s 8; Mental Capacity Act 2005, ss 1 2(5). In some Australian states, legislation similarly provides minors with the right to consent to their own medical treatment; for example, Minors (Property and Contracts) Act 1970 (NSW) s 49; Consent to Medical Treatment and Palliative Care Act 1995 (SA) s 6.

93 Children Act 1989 s 2. ‘Parental responsibility’ means ‘all the rights, duties, powers, responsibilities and authority which by law a parent of a child has in relation to the child and his property’, Children Act 1989, s 3(1). This has been held to include the ability to make decisions for a child in accordance with the child’s best interests: see, for example, *In Re Z (A Minor) (Freedom of Publication)* [1007] Fam 1, 25 *per* Ward LJ.

94
*Bell* (n 6) [47].

95
*Re Kelvin* (n 24) [164].

96 AB v CD (n 8) [114].

97 ibid [72]–[99], [121].

98
*Bell* Court of Appeal (n 9) [4].

99 ibid [86].

100 ibid [77]–[80].

101 ibid [32]–[38], [61]–[65].

102 [2005] EWCA Civ 1003

103 ibid [21]–[22].

104
*Bell* Court of Appeal (n 9) [86].

105 ibid [80].

106 ibid [89].

107 See (n 26).

108 Note that some of the reasoning underpinning this conclusion is discussed further below, in relation to the view that the treatment for GD should be regarded as ‘experimental’.

109
*Bell* (n 6) [135].

110 ibid.

111 There is a body of literature that examines the issue of whether it is appropriate for childhood GD to be included and/or retained in the diagnostic criteria for psychiatric conditions, such as the DSM 5. See, for example, Sam Winter and others, ‘The Psycho-Medical Case against a Gender Incongruence of Childhood Diagnosis’ (2016) 3(5) The Lancet Psychiatry 404; Sam Winter, ‘Gender Trouble: The World Health Organization, the International Statistical Classification of Diseases and Related Health Problems (ICD)-11 and the Trans Kids’ (2017) 14(5) Sexual Health 423.

112
*Bell* (n 6) [135] (emphasis added).

113 It is important to acknowledge that Nicholson CJ’s reasoning here was focused on determining whether treatment for the condition fell within the majority reasoning in *Marion’s Case*, which expressed the view that *therapeutic* treatment is treatment aimed at addressing ‘disease in or malfunctioning of organs’; that is, a *physical* condition. Hence, the classification of the treatment as ‘non-therapeutic’ was significant for this purpose. See *Marion’s Case* (n 12) (Mason CJ, Dawson, Toohey and Gaudron JJ) 250.

114
*Re Alex* (n 26) [195].

115
*Bell* (n 6) [135] (emphasis added).

116
*Bell* Court of Appeal (n 9) [92].

117
*Re Kelvin* (n 24) [141].

118
*PD v SD* [2015] EWHC 4103 (Fam).

119 There were additional circumstances that required the court to consider whether the minor’s parents would be precluded from accessing information regarding his decisions and treatment for GD.

120
*AB v CD* (n 8) [100]–[102].

121 ibid [44].

122 ibid [66].

123 See the conclusions reached by the High Court in *Bell* (n 6) [134]–[135].

124
*Re Jamie* (n 33) [67].

125
*Re Kelvin* (n 24) [156].

126 ibid [156] (Transcript referenced: “Transcript, 21 September 2017, p 66, l 20–23”).

127 Sandra Duffy, ‘Puberty Blockers Ruling will have a Chilling Effect’ *Scottish Legal News* (3 December 2020) <https://www.scottishlegal.com/article/dr-sandra-duffy-puberty-blockers-ruling-will-have-chilling-effect> Accessed 14 July 2021, cited by Moreton (n 7) 6.

128
*Re Kelvin* (n 24) [158]–[160].

129 ibid [19].

130
*Re Kelvin* (n 24) [23], where the Full Court specifically notes that the risks include a risk to life from inappropriate dosage as well as a risk of transmission of blood-borne viruses if the administration of medication/hormones occurs with the use of shared needles and syringes.

131
*AB v CD* (n 8) [97].

132 ibid.

133
*Re Imogen* (n 11).

134 ibid [4].

135 ibid [57].

136 Lisa Littman, ‘Parent Reports of Adolescents and Young Adults Perceived to Show Signs of Rapid Onset Gender Dysphporia’ (2018) 13 PLoS One 8.

137
*Re Imogen* (n 11) [149]–[160].

138 ibid [224]–[230].

139 The judgment notes that the Federal Minister for Health wrote to the RACP seeking advice on the treatment of GD in children and adolescents in Australia, and the RACP responded on 5 March 2020: ibid [161].

140 ibid [163].

141
*Bell* Court of Appeal (n 9) [31].

142 ibid [68].

143
*Bell* (n 6) [138].

144 ibid [126], [133].

145
*Gillick* (n 32) 174.

146 ibid 189.

147 ibid.

148
*Bell* Court of Appeal (n 9) [74].

149 ibid.

150 ibid.

151 ibid [80]–[81].

152 ibid [85].

153 ibid [86].

154 ibid [93].

155
*Re Alex* (n 26) [173].

156 An example of the reliance on medical evidence can be seen in *Re Shay* [2016] FamCA 998 and *Re Dallas* [2016] FamCA 1131.

157 Because the details of the applicant, their family and treating practitioners are confidential, the opportunity to directly analyse the evidence (which contains the full reasons as to why the medical practitioners determine as they do), which is most commonly presented in the form of affidavit, is limited. Engagement with the evidence is therefore only possible in those cases where the judge has cited the relevant views of the expert(s).

158
*Re Spencer* [2014] FamCA 310; *Re Colin* [2014] FamCA 449; *Re Dylan* [2014] FamCA 969; *Re Isaac* [2014] FamCA 1134; *Re Leo* [2015] FamCA 50; *Re Xanthe* [2015] FamCA 116; *Re Jordan* [2015] FamCA 175; *Re Ashley* [2015] FamCA 373; *Re Darcey* [2015] FamCA 409; *Re Christopher* [2015] FamCA 454; *Re Jamie* [2015] FamCA 455; *Re Dale* [2015] FamCA 473; *Re Janson* [2015] FamCA 499; *Re Julian* [2015] FamCA 562; *Re Flynn* [2015] FamCA 629; *Re Kate* [2015] FamCA 705; *Re Karsen* [2015] FamCA 733; *Re Drew* [2015] FamCA 784; *Re Sasha* [2015] FamCA 785; *Re Marley* [2015] FamCA 878; *Re Cameron* [2015] FamCA 1113; *Re Martin* (n 65); *Re Jacinta* [2015] FamCA 1196; *Re Alice* [2015] FamCA 1225; *Re Chloe* [2015] FamCA 1226; *Re Logan* [2016] FamCA 87; *Re George* [2016] FamCA 136; *Re Marco* [2016] FamCA 187; *Re Emery* [2016] FamCA 240; *Re Lincoln* [2016] FamCA 267; *Re Harley* [2016] FamCA 334; *Re Sara* [2016] FamCA 405; *Re Tara* [2016] FamCA 406; *Re Oliver* [2016] FamCA 423; *Re Jerry* [2016] FamCA 437; *Re Gabrielle* [2016] FamCA 470; *Re Celeste* [2016] FamCA 503; *Re Mackenzie* [2016] FamCA 610; *Re Quinn* [2016] FamCA 617; *Re Darryl* [2016] FamCA 720; *Re Jason* [2016] FamCA 772; *Re Tony* [2016] FamCA 936; *Re Shay* (n 156); *Re Lincoln (No 2)* [2016] FamCA 1071; *Re Lucas* (n 66); *Re Dallas* (n 156); *Re Anita* [2016] FamCA 1137; *Re Jan* [2016] FamCA 1171; *Re Matthew* [2017] FamCA 74; *Re Kelvin* (n 70); *Re Kaitlin* [2017] FamCA 83; *Re Daniel* [2017] FamCA 155; *Re Quinn (No 2)* [2017] FamCA 162; *Re Mitchell* [2017] FamCA 185; *Re Desi* [2017] FamCA 203; *Re Jaden* [2017] FamCA 269; *Re Ashton* [2017] FamCA 333; *Re Chelsea* [2017] FamCA 389; *Re Pat* [2017] FamCA 418; *Re Nadia* [2017] FamCA 526; *Re Brittany* [2017] FamCA 527; *Re Benjamin* [2017] FamCA 528; *Re Corey* [2017] FamCA 595; *Re Julian* [2017] FamCA 621; *Re Blaine* [2017] FamCA 647; *Re Dana* [2017] FamCA 686; *Re Ryan* [2017] FamCA 821; *Re Eddie* [2017] FamCA 822; *Re Frances* [2017] FamCA 904; *Re Sage* [2017] FamCA 910; *Re Adrian* [2017] FamCA 957; *Re Hudson* [2017] FamCA 938; *Re Rae* [2017] FamCA 958; *Re Andy* [2017] FamCA 966; *Re Bobbie* [2017] FamCA 974; *Re Kendall* [2017] FamCA 999; *Re Kelvin (No 2)* [2017] FamCA 1000; *Re Eddie (No 2)* [2017] FamCA 1001; *Re Elliott* [2017] FamCA 1008.

159
*Re Spencer* (n 158); *Re Colin* (n 158); *Re Isaac* (n 158); *Re Leo* (n 158); *Re Xanthe* (n 158); *Re Ashley* (n 158); *Re Darcey* (n 158); *Re Christopher* (n 158); *Re Jamie* (n 158); *Re Dale* (n 158); *Re Janson* (n 158); *Re Julian* [2015] (n 158); *Re Flynn* (n 158); *Re Kate* (n 158); *Re Drew* (n 158); *Re Sasha* (n 158); *Re Martin* (n 65); *Re Jacinta* (n 158); *Re Logan* (n 158); *Re George* (n 158); *Re Marco* (n 158); *Re Emery* (n 158); *Re Lincoln* (n 158); *Re Harley* (n 158); *Re Sara* (n 158); *Re Tara* (n 158); *Re Oliver* (n 158); *Re Gabrielle* (n 158); *Re Celeste* (n 158); *Re Mackenzie* (n 158); *Re Quinn* (n 158); *Re Darryl* (n 158); *Re Jason* (n 158); *Re Tony* (n 158); *Re Shay* (n 156); *Re Lincoln (No 2)* (n 158); *Re Dallas* (n 156); *Re Lucas* (n 66); *Re Anita* (n 158); *Re Matthew* (n 158); *Re Kelvin* (n 70), (n 158); *Re Daniel* (n 158); *Re Quinn (No 2)* (n 158); *Re Mitchell* (n 158); *Re Desi* (n 158); *Re Jaden* (n 158); *Re Ashton* (n 158); *Re Chelsea* (n 158); *Re Pat* (n 158); *Re Nadia* (n 158); *Re Brittany* (n 158); *Re Benjamin* (n 158); *Re Corey* (n 158); *Re Julian* [2017] (n 158); *Re Blaine* (n 158); *Re Dana* (n 158); *Re Ryan* (n 158); *Re Eddie* (n 158); *Re Frances* (n 158); *Re Sage* (n 158)*; Re Adrian* (n 158); *Re Andy* (n 158); *Re Kendall* (n 158).

160
*Re Alice* (n 158); *Re Chloe* (n 158).

161
*Re Dylan* (n 158); *Re Jordan* (n 158); *Re Cameron* (n 158); *Re Karsen* (n 158); *Re Marley* (n 158); *Re Jerry* (n 158); *Re Jan* (n 158); *Re Hudson* (n 158); *Re Bobbie* (n 158).

162
*Re Karsen* (n 158) [30].

163 ibid.

164 See *Re Marley* (n 158) [40].

165
*Re Hudson* (n 158) also provides an example of a case where the minor is deemed not *Gillick* competent to make a decision, and the judge explains the reasoning for this.

166
*Re Isaac* (n 158); *Re Gabrielle* (n 158).

167 See *Re Anita* (n 158); *Re Quinn* (n 158). The latter case relates to a decision about Stage 3 treatment.

168 See *Re Shay* (n 156); *Re Dallas* (n 156).

169 See (n 158).

170 See (n 158).

171 ibid [23].

172 It should be noted that item six was not explicitly listed in the requirements nor directly analysed in the cases of *Re Lincoln (No 2)* (n 158) and *Re Elliott* (n 158), but was listed in *Re Imogen* (n 11) [184]–[185].

173 This list has evolved from the decisions in *Re Lincoln (No 2)* (n 158); *Re Elliott* (n 158); *Re Imogen* (n 11).

174
*Re Elliott* (n 158) [23].

175
*Re Dylan* (n 158); *Re Jordan* (n 158); *Re Cameron* (n 158); *Re Karsen* (n 158); *Re Marley* (n 158); *Re Jerry* (n 158); *Re Jan* (n 158); *Re Hudson* (n 158); *Re Bobbie* (n 158).

176 See *Re Karsen* (n 158); *Re Marley* (n 158).

177 See *AB v CD* (n 8) [122].

178 Beth A Clark and Alice Virani, ‘This Wasn’t a Split-Second Decision: An Empirical Ethical Analysis of Transgender Youth Capacity, Rights, and Authority to Consent to Hormone Therapy’ (2021) 18 Journal of Bioethical Inquiry 151, 153.

179 ibid 159.

180
*Bell* (n 6) [134]–[137].

181 ibid [136].

182
*Bell* Court of Appeal (n 9) [26]–[29], [34].

183 ibid [27].

184
*Re Alex* (n 26) [186].

185
*Re Brodie (Special Medical Procedures)* [2008] FamCA 334 [38].

186
*Re Jamie* (n 47). This was in line with the thinking of other first-instance decisions decided around the same time, by Family Court judges, including *Re Lucy* [2013] FamCA 518; *Re Sam and Terry* (n 47).

187
*Re Kelvin* (n 24) [202]–[207]. Note, however, that the minority in *Re Kelvin* disagreed with the earlier decision in *Re Jamie*, and said that the factors of irreversibility and the seriousness of the treatment alone did not engage the authority of *Marion’s Case* for the determination that Stage 2 treatment is a special medical procedure.

188 World Professional Association for Transgender Health (n 77).

189 ibid 18

190 ibid 19, emphasis added.

191 See the *Australian standards of care and treatment guidelines for trans and gender diverse children* on this point, which set out criteria for the commencement of gender affirming hormone treatment. Telfer and others (n 2) 24.

192
*Bell* Court of Appeal (n 9) [64].

193 Telfer and others (n 2) 11; World Professional Association for Transgender Health (n 77) 14.

194
*Re Kelvin* (n 24) [162].

195 Due to the focus of this article, which is centred on the comparison with the Australian perspective rather than an analysis of the approach to capacity per se, the opportunity to fully analyse the arguments surrounding the approach to capacity adopted in the recent English cases is limited. However, it is noted that there is a need to consider whether the approach adopted by the High Court in *Bell* accords with a functional approach to capacity, which is typically adopted in most of the legislative frameworks relevant to capacity in both Australia and England and Wales. See Moreton (n 7) for an overview of some of the issues with the High Court’s interpretation and application of the test for *Gillick* competency.

196
*AB v CD* (n 8) [97]–[99].

197
*Marion’s Case* (n 12).

198
*Re Baby A* [2008] FamCA 417 [16].

199
*Bell* (n 6) [134].

200 ibid.

201 ibid.

202 ibid.

203 ibid [148].

204 ibid [134].

205 ibid [74].

206
*Practice Guidance (Court of Protection: Serious Medical Treatment)* [2020] 1 WLR 641: *Bell* (n 6) [95]–[96].

207 ibid.

208
*Bell* (n 6) [120]–[121].

209
*AB v CD* (n 8) [96].

210
*Re Jamie* (n 33) [60] and [84].

211
*Re Kelvin* (n 24) [162].

212
*Re Imogen* (n 11) [162].

213 See Jowett and Kelly (n 86).

## Notes

I would like to thank the two anonymous reviewers who provided very positive and helpful comments on an earlier draft of this article. I would also like to thank one of the Editors of this Journal, who provided very helpful and constructive feedback on an earlier draft of this article.

